# Satellite telemetry reveals complex mixed movement strategies in ibis and spoonbills of Australia: implications for water and wetland management

**DOI:** 10.1186/s40462-024-00515-4

**Published:** 2024-11-26

**Authors:** Heather M. McGinness, Luke R. Lloyd-Jones, Freya Robinson, Art Langston, Louis G. O’Neill, Shoshana Rapley, Micha V. Jackson, Jessica Hodgson, Melissa Piper, Micah Davies, John M. Martin, Richard Kingsford, Kate Brandis, Veronica Doerr, Ralph Mac Nally

**Affiliations:** 1 CSIRO Environment, GPO Box 1701, Canberra, ACT 2601 Australia; 2 CSIRO Data61, Brisbane, QLD 4102 Australia; 3 CSIRO Health and Biosecurity, Canberra, ACT 2601 Australia; 4 CSIRO Agriculture, Canberra, ACT 2601 Australia; 5 Royal Botanic Gardens Sydney, Sydney, NSW 2000 Australia; 6 University of New South Wales, Sydney, NSW 2052 Australia; 7 University of Canberra, Canberra, ACT 2615 Australia; 8 School of Biosciences, The University of Melbourne, Parkville, 3052 Australia; 9 Fenner School of Environment and Society, The Australian National University, Canberra, ACT 2600 Australia

**Keywords:** Environmental water, Satellite telemetry, Foraging, Nomadic, Conservation management

## Abstract

Waterbird population and species diversity maintenance are important outcomes of wetland conservation management, but knowledge gaps regarding waterbird movements affect our ability to understand and predict waterbird responses to management at appropriate scales. Movement tracking using satellite telemetry is now allowing us to fill these knowledge gaps for highly mobile waterbirds at continental scales, including in remote areas for which data have been historically difficult to acquire. We used GPS satellite telemetry to track the movements of 122 individuals of three species of ibis and spoonbills (*Threskiornithidae*) in Australia from 2016 to 2023. We analysed movement distances, residency periods and areas, and foraging-site fidelity. From this we derived implications for water and wetland management for waterbird conservation. This is the first multi-year movement tracking data for ibis and spoonbills in Australia, with some individuals tracked continuously for more than five years including from natal site to first breeding attempt. Tracking revealed both inter- and intra-specific variability in movement strategies, including residency, nomadism, and migration, with individuals switching between these behaviours. During periods of residency, areas used and distances travelled to forage were highly variable and differed significantly between species. Sixty-five percent of identified residency areas were not associated with wetlands formally listed nationally or internationally as important. Tracking the movements of waterbirds provides context for coordinated allocation of management resources, such as provision of environmental water at appropriate places and times for maximum conservation benefit. This study highlights the geographic scales over which these birds function and shows how variable waterbird movements are. This illustrates the need to consider the full life cycle of these birds when making management decisions and evaluating management impacts. Increased knowledge of the spatio-temporal interactions of waterbirds with their resource needs over complete life cycles will continue to be essential for informing management aimed at increasing waterbird numbers and maintaining long-term diversity.

**Supplementary Information:**

The online version contains supplementary material available at 10.1186/s40462-024-00515-4.

## Introduction

Waterbirds are highly mobile, with many species conducting long-distance movements of hundreds or thousands of kilometres in days or weeks [1–3]. Understanding these movements is key to effective management of waterbird populations [4], particularly since rates of wetland habitat loss are increasing due to water harvesting and climate drying [4–7]. Maintaining waterbird diversity and habitats are often important goals for water and wetland managers worldwide [8–10]. However, there are knowledge gaps that affect our ability to manage habitats and to predict waterbird responses at local, national and global scales. One of the most critical gaps is knowledge of waterbird movement behaviour and variability, particularly for inland species [4, 11–13].

Waterbird movements can be complex, occurring along a continuum of variation where the behaviour of any one species or individual may be classified variously as: (1) Residency, including ‘sedentary movements’, ‘central-place movements’, and ‘commuting’, where individuals remain in one area,, usually returning to favoured locations between relatively short-distance foraging trips, and sometimes displaying territoriality (including during breeding periods); (2) Nomadism, including ‘facultative movements’ where individuals move different distances and directions irregularly and seem to generally be responding opportunistically to resource availability, including ‘fugitive movements’ in response to disruption or disturbance of resources, individuals or flocks; or (3) Migration, sometimes called ‘obligatory migration’ or ‘seasonal migration’, where individuals regularly move relatively long distances predictably between particular locations and in consistent directions, usually seasonally or annually [14–16]. Adding to this complexity, individuals can show plasticity in their movement strategies over time [17].

Advances in telemetry are revealing complexities in movement patterns within and among individuals and species, including for example ‘partial migration’, where residency, nomadism or migration strategies may each be employed within a species by different individuals or by an individual at different times [18, 19] It has been suggested that there is greater variation in movement strategies in the southern hemisphere compared to the northern hemisphere due to greater variability in climatic factors [20, 21]. However, there are relatively few satellite telemetry studies tracking southern hemisphere species, especially inland waterbirds, and those few have usually involved relatively small numbers of individuals [12, 22–24]. Moreover, understanding of nomadism is relatively limited compared to that for migration and residency [16, 19, 25].

Movement ecology is often poorly understood even for common and conspicuous taxa that are the focus of significant management investment. Examples include ibis, spoonbills, egrets, and herons that nest in large aggregations, often in Ramsar sites, in response to specific environmental conditions such as flooding [11, 26, 27]. Despite extensive leg-banding and other marking programs, resighting or recovery data are usually limited for these taxa. For example, < 0.8% of ibis and spoonbills banded in Australia have been resighted or had their bands found > 3 months after banding, post-dispersal from natal or nest sites [28]. Taxa such as these are of particular interest for wetland and waterbird management and policy makers because they often nest in areas that are susceptible to adverse effects of environmental change; consequently, their habitats and populations are the subject of intensive conservation management [29–33]. For species dependent on surface water, management can include the allocation of environmental water or ‘environmental flows’, to provide the quantity, timing, and quality of freshwater flows and levels necessary to sustain aquatic ecosystems [34]. Wetlands and other areas that receive environmental water (actively or passively), or that may be inundated by natural flooding if key constraints are overcome, are colloquially termed the ‘managed floodplain’ [35]. Understanding waterbird movements at fine spatial and temporal scales can maximise the efficacy of environmental water application, by guiding where and when to provide water and for how long.

Australia has breeding populations of three ibis species and two spoonbill species in the Threskiornithidae family: Straw-necked ibis (*Threskiornis spinicollis*); Australian white ibis (*Threskiornis molucca*); glossy ibis (*Plegadis falcinellus*); royal spoonbill (*Platalea regia*); and yellow-billed spoonbill (*Platalea flavipes*). These species nest in aggregations of up to hundreds of thousands of birds when conditions are good, while in poor conditions they may not nest at all. Inland, these species are experiencing reduced breeding event frequency, size, and success, including mass nest abandonment events due to prematurely falling water levels [36–38]. The birds are dependent on wetland inundation for breeding, but the degree of breeding-site fidelity among individuals or groups is not well understood, even within the relatively well-studied breeding sites of the Murray-Darling Basin in south-eastern Australia, which is thought to be the core breeding area for these species, with 46% of aggregate-nesting wetlands in Australia [39]. Some breeding sites are used every year by the same species, but it is not known if it is the same individuals revisiting each time.

There are major knowledge gaps about movements outside of breeding events. While these birds are known to be capable of moving at continental scales (many 100 s – 1000 s km) within a few months [27, 28, 39, 40], some authors have suggested there may be regional sub-populations and seasonal migrations with site fidelity [41, 42]. Leg-banding data suggest individuals that breed in eastern Australia mostly remain in eastern Australia and rarely move west; most recorded displacements of leg-banded birds have been from the south-east to the north-east [28, 39, 40, 43]. Seasonal fluctuations in local abundance along with observations of flocks in flight suggest that east coast areas are important winter and drought refuge locations, while inland wetlands of the Murray-Darling Basin are important breeding sites [28, 39, 41]. However banding recoveries do not suggest any relationships among bird age, season, and movement distances or directions [28, 39, 40].

There has been very limited direct movement tracking using telemetry for ibis and spoonbill species in Australia. No royal spoonbills, yellow-billed spoonbills, or glossy ibis have been tracked prior to this study. In February 2000, two straw-necked ibis were fitted with satellite transmitters in the Macquarie Marshes in New South Wales (NSW) and tracked for c. two months [27]. Of these two, one bird flew 1438 km north of the capture site within a month, while the second bird remained within 200 km of the capture site [27]. Tracking of Australian white ibis movements has largely focused on breeding sites in urban and suburban coastal environments, rather than natural inland wetlands [44–46].

For conservation and management, important knowledge gaps remain at the continental scale about movement patterns, movement variability, movement rates and movement timing for these species. At local to regional scales, questions such as how far birds travel to find food are also relevant for managers of waterbird populations and habitats. Answering these questions would assist water and wetland managers to identify key habitats associated with movements and to understand better the places, scales and times at which resources are required. This should in turn improve capacity to target land and water management actions (such as strategic watering and drying of wetlands), evaluate progress, and predict future outcomes for waterbirds.

Over seven years, we used Global Positioning System (GPS) satellite telemetry to track the movements of three species in the Threskiornithidae family – straw-necked ibis, Australian white ibis, and royal spoonbills. We aimed to address knowledge gaps regarding intra and interspecific variation in movement strategies of these species post-dispersal from breeding sites and consider consequent implications for water, wetland and waterbird management.

## Methods

### Transmitter deployment

Straw-necked ibis (‘SNI’) was chosen as the primary species for transmitter deployment because it is a focal species for Australian wetland and water managers that nests in large numbers in major inland wetlands managed with environmental water. Two other species that frequently nest and forage with SNI were also tracked, in smaller numbers, to explore potential differences among species: the royal spoonbill (‘RSB’) and the Australian white ibis (‘AWI’).

**Fig. 1:**
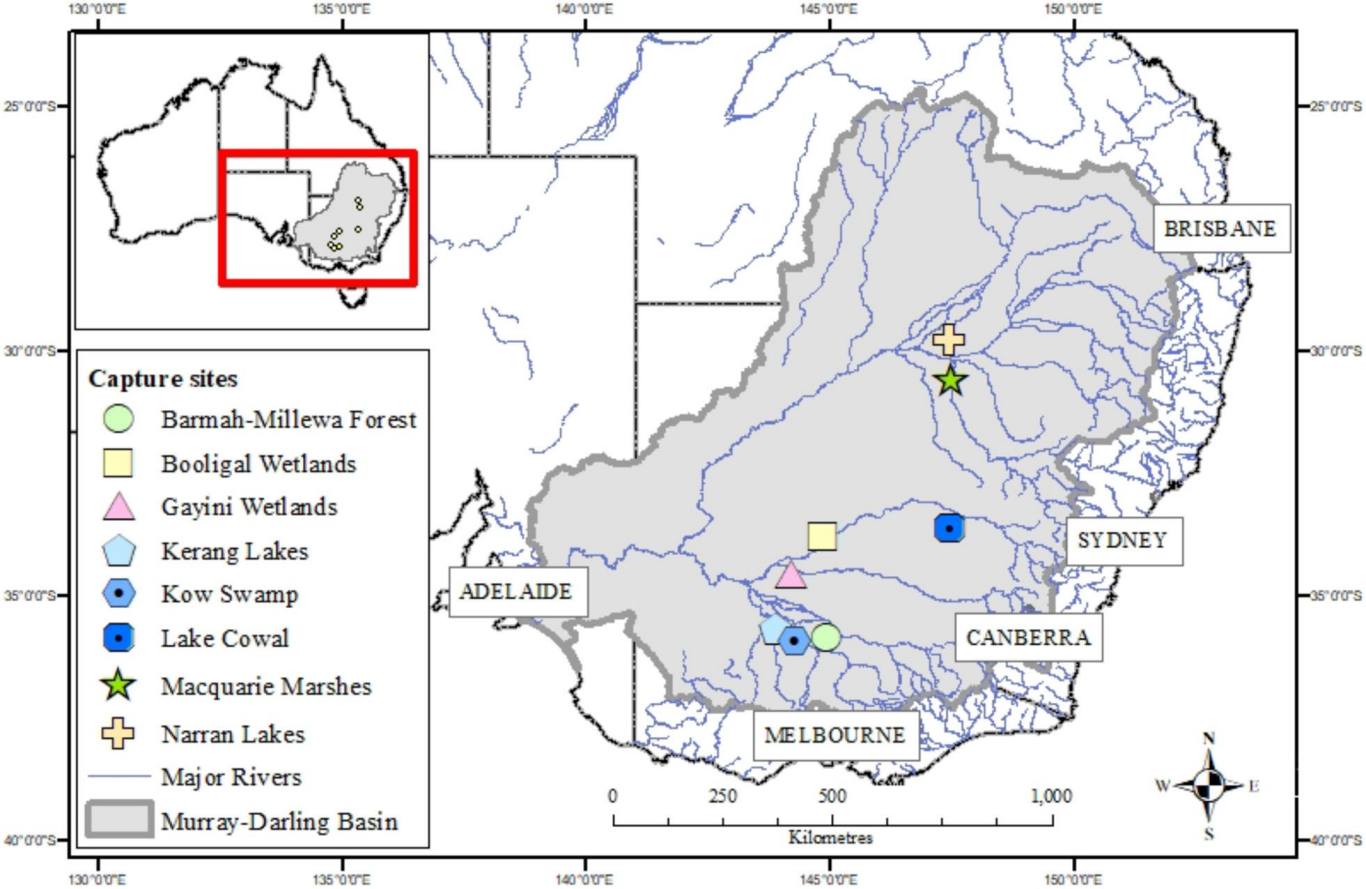
Sites at which straw-necked ibis, Australian white ibis and royal spoonbill were fitted with transmitters, south-eastern Australia

Transmitters were deployed at eight breeding sites within the Murray-Darling Basin in south-eastern Australia (Fig. 1) between 2016 and 2023. The Murray-Darling Basin (‘the Basin’) is ≥ 10^6^ km^2^ and is a primary focus for intensive water management and water policy reform in Australia [47], while facing significant challenges from climate change [48, 49]. It is mostly temperate in the south, semi-arid in the west, and sub-tropical in the north. The hydrology of the Basin is highly variable spatially and temporally, with substantial transmission losses through long lowland rivers and high evapotranspiration rates [50]. Consequently, many of the Basin's rivers flow intermittently, with natural longitudinal and lateral disconnections, while in wet years, floods move slowly and spread out to inundate extensive floodplain and wetland areas [50]. Many of the 16 internationally significant wetlands in the Basin are located in these large floodplains and dependent on flooding including Ramsar-listed wetlands [51] and some of the most important aggregate-nesting ibis and spoonbill breeding sites on the continent [37, 52].

We captured birds either by hand, or with leg-nooses, or with a net launcher, depending on the site and environment. Birds were placed in large clean calico bags for weighing and other measurements. Juveniles were distinguished from adults by plumage, skin, leg, and size differences. Transmitters were attached as a ‘backpack’ using Teflon ribbon or Spectra ribbon (Bally Ribbon Mills™) harnesses, fitted either as wing-loops with a join at the keel (SNI and AWI and some RSB), or as leg-loops (most RSB). Harness design was based on designs used in other species [53–56] modified and improved over time, with different types of weak links used in different years. Transmitters weighed 12–40 g, ranging from < 1% to 5% of bird bodyweight [57].

Solar-powered GPS transmitters with a fix accuracy of 15–26 m were used, with data sent through either the Argos satellite network (Geotrak units) or the 3G network (Ornitela and Druid units). The frequency of fixes ranged from every min to every 6 h, depending on the transmitter type and programmed schedule. This was handled in analyses, with interpolation or down-scaling applied when appropriate (see below).

### Analyses

Analyses were done in R version 4.3.1. Data for nesting adults, for adults and juveniles still within breeding sites post-capture but pre-dispersal, and for birds tracked for < 30 days were removed, to ensure results reflected longer-term movement statistics and patterns. Summary statistics were calculated for each species and age class for tracking duration, distance travelled between nocturnal roosts and diurnal foraging sites (maximum distance travelled from roost per day), roost-shift distances (midnight fix to midnight fix displacements), and cumulative distance travelled per hour (km h^−1^), per 24 h (km d^−1^) and per year (km yr^−1^). Statistics for distances travelled were calculated separately for calendar months to account for seasonal differences, by species and age class. Permutation tests were used to asses differences in distributions between groups of interest e.g., species and age using the ‘*percentileTest*’ and ‘*pairwisePercentileTest*’ functions with 1000 replicates in the R package ‘*rcompanion*’ [58, 59].

#### Residency analyses

For analyses of residency periods and areas, a down-scaled dataset with each bird placed on a common 6-hourly grid was used. Periods of residency were identified using the semivariance function (SVF) approach of Fleming et al. [60] using the tools in the ‘Continuous Time Movement Models’ R package ‘*ctmm’* [61]. The SVF measures the variability in the distances between pairs of locations as a function of the time lag between those pairs and is calculated over all possible time lags up to the maximum. This method was initially trialled on both the entire movement track of each individual and various temporal sub-sections of the movement track, but sections longer than a few months rarely passed the residency tests required for estimations. Ultimately, monthly segments were chosen as the longest frequently resident sections of the telemetry tracks to explore changes in behaviour through time and to allow evaluation of seasonal patterns.

To classify months of resident behaviour, a within-month Ornstein–Uhlenbeck-including-foraging (OUF) model was fitted to the empirical variogram using functions in the R package ‘*ctmm’* [61]. We chose the OUF model because it is frequently the best fitting model for resident behaviour modes [60], and the scale of our data (~ 1300 total months to analyse across all tracked individuals) made model comparison impractical. Automated first- and second-order derivative checks were performed on the fitted OUF function to determine whether the function reached an asymptote within each month. Reaching an asymptote defined the months for each individual in which the bird was resident.

The spatial area of habitat used during each period of temporary residency was calculated using the autocorrelated kernel density estimation (AKDE) method, fitted using the R package ‘*ctmm’* [62]. First, for each month that each bird was resident, the within-month maximum likelihood estimate of the 95% contour of the kernel density was defined and the area computed. Then, to determine whether more than one month should be included in the estimation of residency area, a test for overlap of residency areas between months of residency was performed within sequential pairs of months of residency using the Bhattacharyya coefficient (BC) method in the R package ‘*ctmm’* [63]. The BC quantifies the overlap between areas and provides a statistical test for whether the overlap is substantial enough to state they are distinct, given model uncertainty [63]. Residency areas were considered to overlap if the lower confidence interval was greater than 0.01 as in Winner et al*.* 2018 [63]. For the sets of months in which the area values overlapped (i.e., exceeded the threshold as determined by the BC test), the AKDE method was rerun to estimate a residency area for a joint time period, which we refer to as a ‘block’. The mean and median over each individual’s discontiguous 95% area blocks were summarised. Differences in residency areas among species or by age class were investigated by testing differences in medians via permutation tests using the ‘*percentileTest’* function in the R package ‘*rcompanion’*.

Once periods of residency had been established as outlined above, we investigated the frequency of residency across individuals, species and age groups. For all birds, the odds of residency vs non-residency for each calendar month were computed from the counts in each of the two classes and the confidence interval from delta method approximation [64] for the odds as a function of the sample proportion estimate i.e., the standard error was computed on the proportion estimate and then mapped to the interval on the odds scale via the delta method. Using the resident/non-resident status over all months for all birds, we computed the odds of being resident as the ratio of the number of resident months to non-resident months. For example, if there were 100 instances of telemetry tracks covering the month of June (which could be multiple instances per bird if they were tracked for multiple years) then an odds of 2 indicates that 66 tracks from June were classified as resident versus 33 as non-resident. This was done for all birds combined, for individual species, and for age groups within species. Differences in the proportion of residency events in season were compared with the two-sample permutation test to compare two proportions implemented using the ‘*twoSamplePermutationTestProportion*’ function in the R package ‘*EnvStats’* [65].

#### Site fidelity

To explore fidelity to foraging areas (locations and periods of time for which individuals roosted and foraged in the same area but were not breeding), sites used during periods of residency were mapped as polygons representing the utilisation distribution area based on the start and end date of each residency period (‘UD’, representing the probabilistic expression of the residency area). The R package ‘*Recurse*’ [66] was used to calculate visitation to each site polygon, in terms of arrival date, departure date, duration of occupancy, duration between visits, and number of distinct visits overall and by month, for each individual.

Residency areas were mapped and characterised according to whether they were associated with: (a) sites listed by the Ramsar Convention on Wetlands of International Importance [51]; (b) sites listed by the Directory of Important Wetlands in Australia [67]; (c) the ‘managed floodplain’, being wetlands and other areas that can receive environmental water (actively or passively) or that may be inundated by natural flooding or high flows if key constraints are overcome [35]; and, (d) historical breeding sites for these and other aggregate-nesting species within the Murray-Darling Basin, which is thought to be the core breeding area for these species and for which breeding site locations are relatively well known and mapped compared to other parts of Australia.

## Results

A total of 194 individuals of the three selected species were fitted with transmitters (119 SNI, 65 RSB, and 10 AWI). The final dataset comprised 122 individuals, 41,619 days of tracking data and 1,240,206 GPS fixes (Table 1), with SNI n = 73; 45 adults and 28 juveniles, RSB n = 42; five adults and 37 juveniles, and AWI n = 7; three adults and four juveniles. The maximum number of days that any individual was tracked was 2233 days. Five adult and two juvenile SNI and two juvenile AWI were tracked for > 3 years (Supplementary Table 1).

**Table 1:** Percentage time (months) classed as resident by species and age group

Species	Age class	% months resident by species and age group
AWI	Adult	58
AWI	Juvenile	49
RSB	Adult	33
RSB	Juvenile	30
SNI	Adult	24
SNI	Juvenile	30

### Distance

**Fig. 2:**
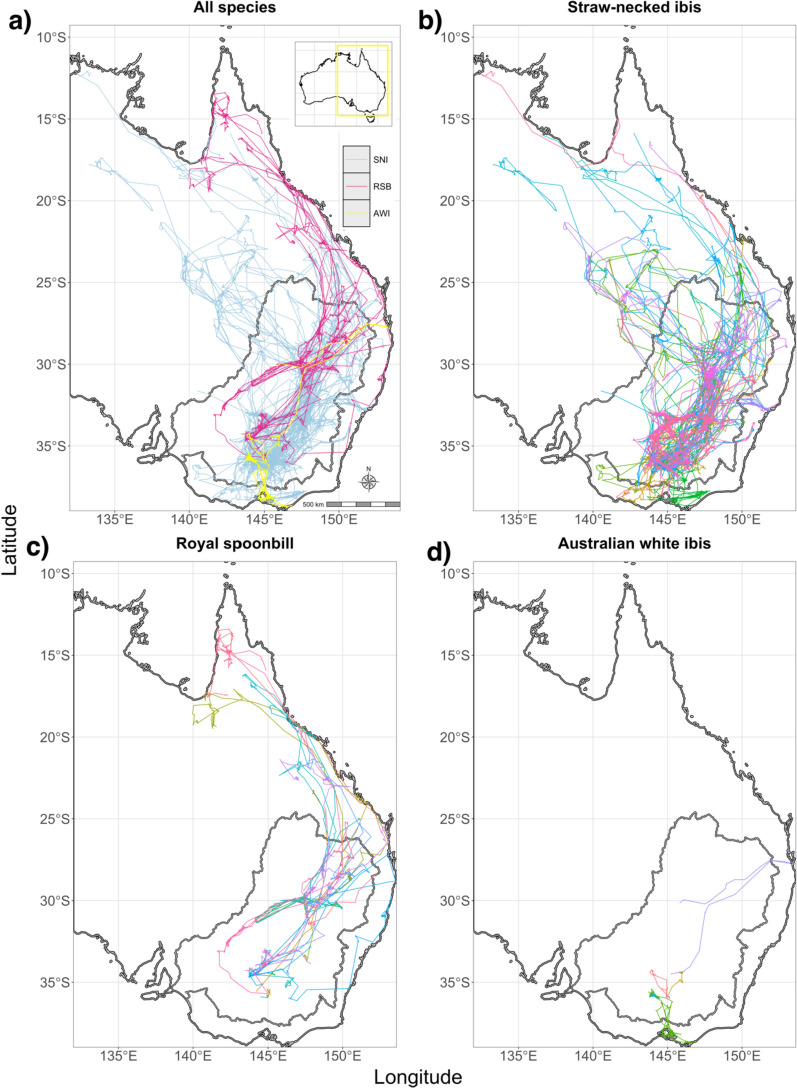
Panels depict: **a** all satellite-tracked SNI, RSB and AWI movements in eastern Australia combined, 2016 – 2024 (*n* = 122) with different colours representing different species; **b** SNI movements (*n* = 73); **c** RSB movements (n = 42); **d** AWI movements (*n* = 7). Within species, colours indicate individuals

**Fig. 3:**
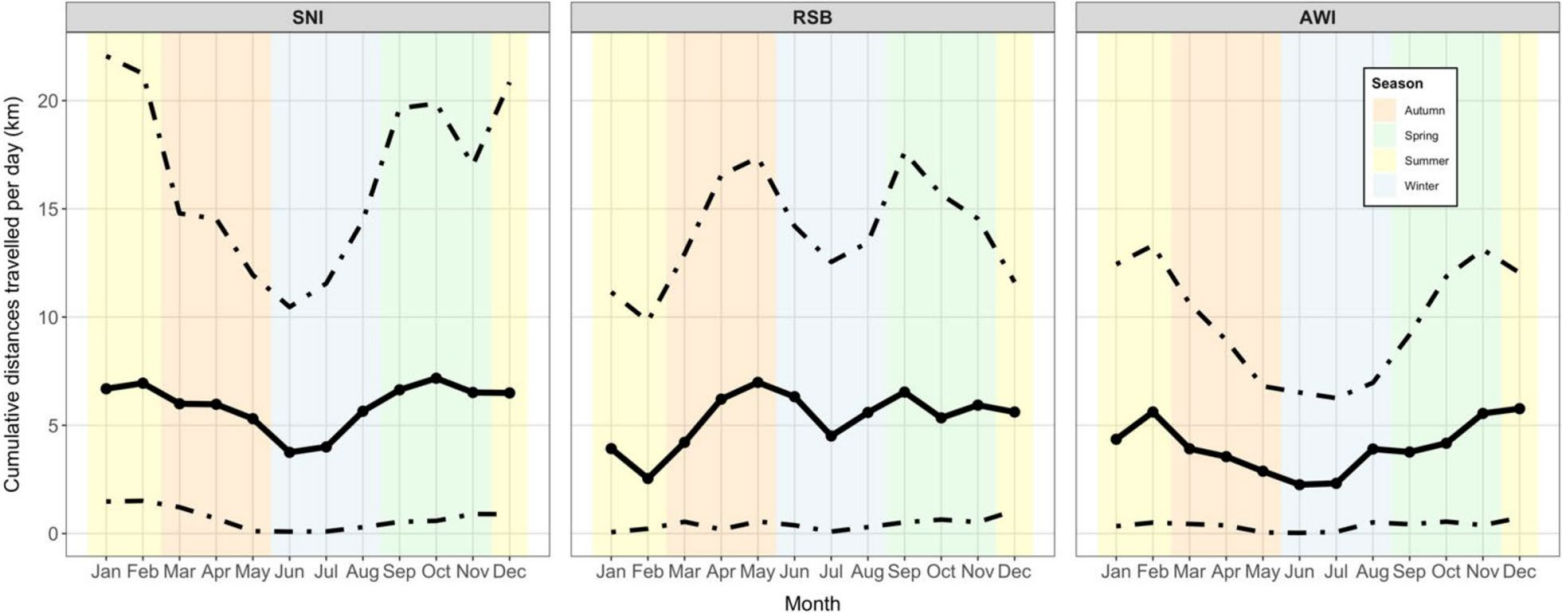
Mean cumulative distance travelled per 24 h, by month and species. Central solid line is the trend in the median values. Dotted lines show the trend in the lower 10th percentile and the upper 80th percentile, which were chosen so that the change in variation can be observed between months, but the median trends are evident (see Supplementary Fig. 2 for full distributions of cumulative distances by month). Shaded regions show the season of the year in the southern hemisphere

Tracked movements spanned almost the whole of eastern mainland Australia (Fig. 2). The longest distance travelled in an hour was 135 km, recorded by a juvenile SNI. The longest cumulative distance travelled within a 24 h period was 857 km, recorded for a juvenile RSB; maximums for SNI and AWI were 662 km and 271 km respectively. The median cumulative distance travelled per 24 h differed between species, with SNI travelling the furthest at 5.9 km d^−1^, AWI travelling 3.9 km d^−1^, and RSB traveling 5.4 km d^−1^ (permutation *P* < 0.001 adjusted for multiple testing from pairwise permutation tests between medians (null hypothesis of no difference in medians) of each species; see Supplementary Fig. 1 for distributions). Cumulative distances travelled per day were lower in the late autumn and early winter months and higher in spring and summer for SNI and AWI; for RSB, mean distances travelled per day were highest in autumn (Fig. 3). Permutation *P*-values between medians and 80th percentiles between seasons within species were < 1e^−6^ (null hypothesis of no difference in median distance travelled between seasons) except for summer and spring comparisons within AWI and SNI. RSB showed evidence for differences in 80th percentiles, except for autumn and spring and summer differences in medians. The 80th percentiles summarise the components of the monthly distribution that are long-distance movements while the medians summarise the central tendency of the heavily right-skewed distributions (see Supplementary Fig. 2 for summaries of full distributions). For those individuals with complete datasets of 365 days in a year (adjusted for removal of nesting periods and missing data), the maximum cumulative distance travelled by an individual was 15,549 km yr^−1^ (an adult SNI).

Species also differed in the distances travelled from roosts to foraging sites (maximum distance travelled from roost per day; permutation test *P* < 0.001). Distances were shortest for AWI (median 1.6 km, mean 3.7 ± 10.7 km) compared to SNI (median 2.1 km, mean 10.8 ± 35.1 km) and RSB (median 2.4 km, mean 8.1 ± 27.9 km; Supplementary Fig. 3 Juvenile SNI travelled shorter distances (median 1.6 km) to forage than did adults (median 2.5 km; *P* < 0.001; Supplementary Table 2).

**Fig. 4:**
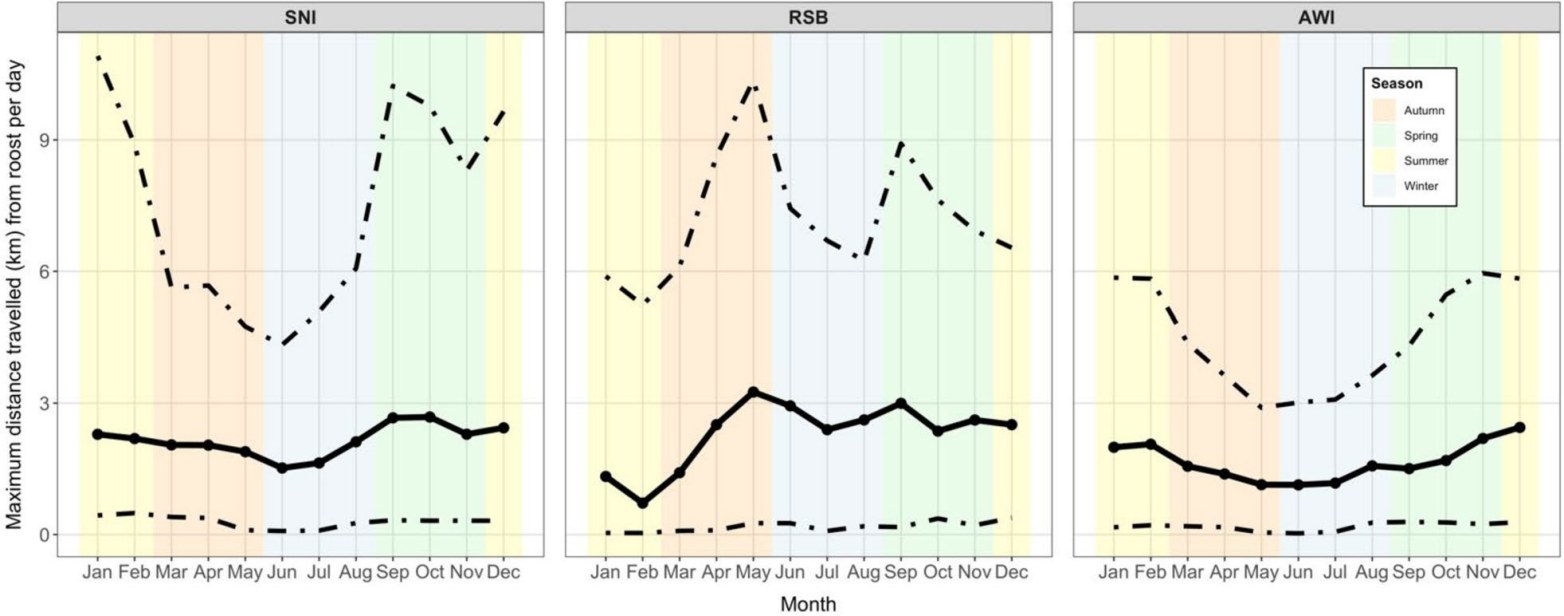
Mean distance travelled to forage from roost site, by species and month. Central solid line is the trend in the median values. Dotted lines show the trend in the lower 10th percentile and the upper 80th percentile, which were chosen so that the change in variance can be observed between months, but the median trend can be visually appreciated (see Supplementary Fig. 4 for full distributions of cumulative distances by month). Shaded regions show the season of the year in the southern hemisphere

Daily distances travelled from the roost differed among months of the year for SNI and RSB but less so for AWI (Fig. 4). SNI travelled further from their roosts in spring and summer months compared to late autumn and early winter months (permutation test for median differences *P* < 1e^−6^ for all season comparisons; 80th percentile permutation test *P* = 0.74 for the summer-spring comparison and 0.04 for the autumn–winter comparison). In contrast, RSB travelled farther to forage in late autumn, and foraged closer to roosts in summer (Fig. 4; permutation values between summer median distance travelled and all other seasons *P* < 1e^−6^).

There was evidence for greater roost-site fidelity for AWI than for the other species, with median roost location shift distances of 0.3 km (mean 2.7 ± 10.7 km), compared to 0.6 for SNI (mean 9.4 ± 35.0 km) and 0.6 for RSB, (mean 7.8 ± 32.5 km; *P* < 0.001). Median roost location shift distances were also shorter for juvenile RSB and SNI (0.55 km and 0.45 km) compared to adults of these species (0.66 km and 0.78 km respectively; permutation *P*- < 1e^−6^).

### Residency areas

**Fig. 5:**
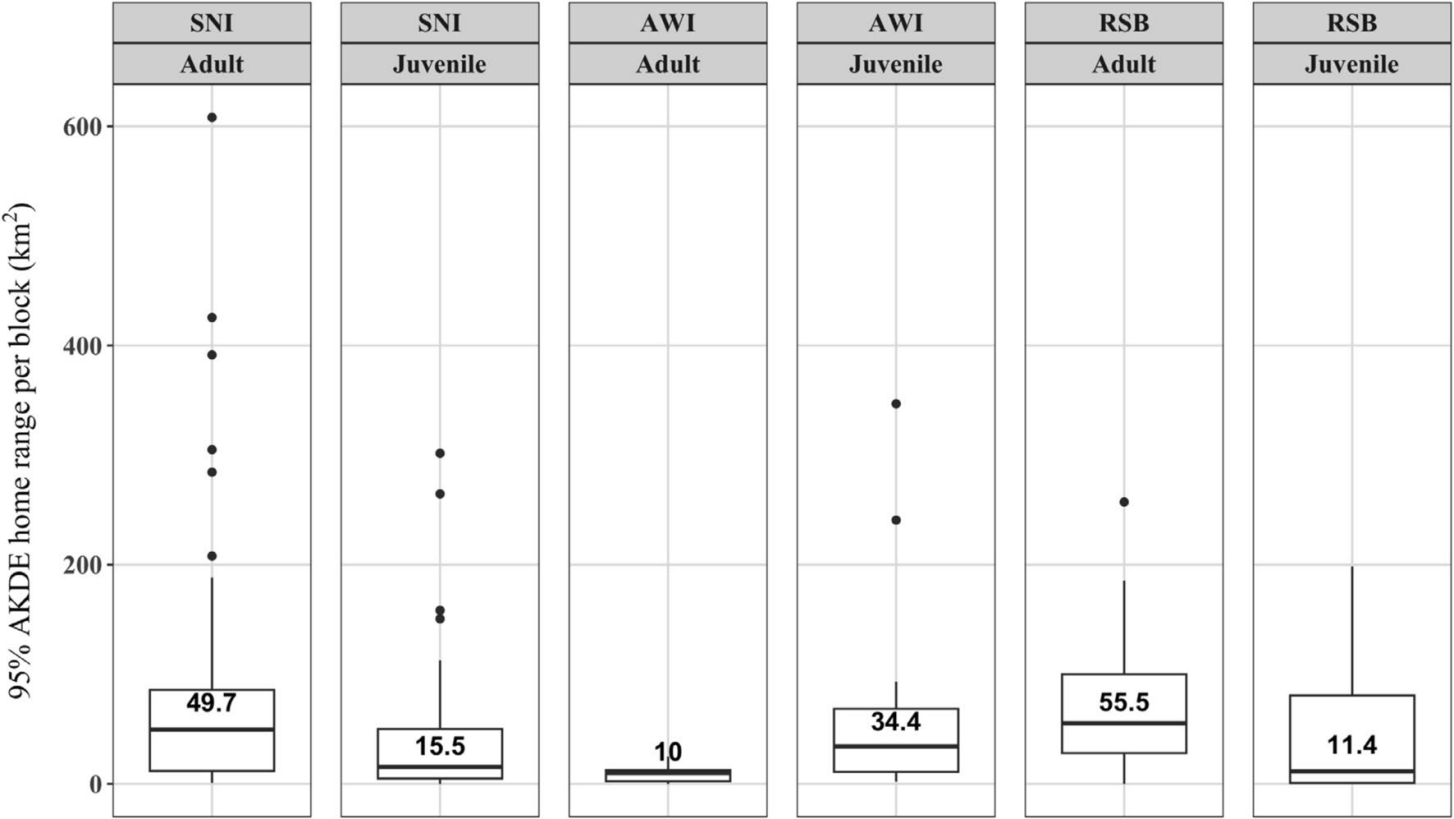
Summary of 95% autocorrelated kernel density estimated (AKDE) residency block area distributions by species and age class. Numbers above boxes correspond to median values

Areas used when resident differed among species for adults. AWI adults had significantly smaller residency areas (median 10.0 km^2^) than SNI and RSB (medians 49.7 and 55.5 km^2^ respectively; Fig. 5; Supplementary Table 3; see permutation test results in Supplementary Fig. 5). Adult SNI and RSB also had larger median residency areas than juveniles of the same species (Fig. 5), but this was only significant for permutation testing of a greater median for adults for SNI (permutation *P* = 0.01, see Supplementary Fig. 6). In contrast, the median residency area of juvenile AWI (34.4 km^2^) was nearly four times greater than that of adult AWI (10.0 km^2^), however there were limited data for AWI, so these observations should be treated with caution (permutation *P* = 0.02, see Supplementary Fig. 6).

### Frequency of residency

Across all species, 25% of individuals had no period of residency in any month according to the criteria detailed above. All individuals with no residency periods were either SNI or RSB (SNI = 19 adults, 4 juveniles, accounting for 31.5% of tracked SNIs; RSB = 6 adults, 1 juvenile, accounting for 16.6% of tracked RSB). In contrast, all tracked AWI had multiple or extended periods of residency. The proportion of months spent resident overall ranged from 24% (adult SNI) to 33% (adult RSB) and 58% (adult AWI; Table 1).

**Fig. 6:**
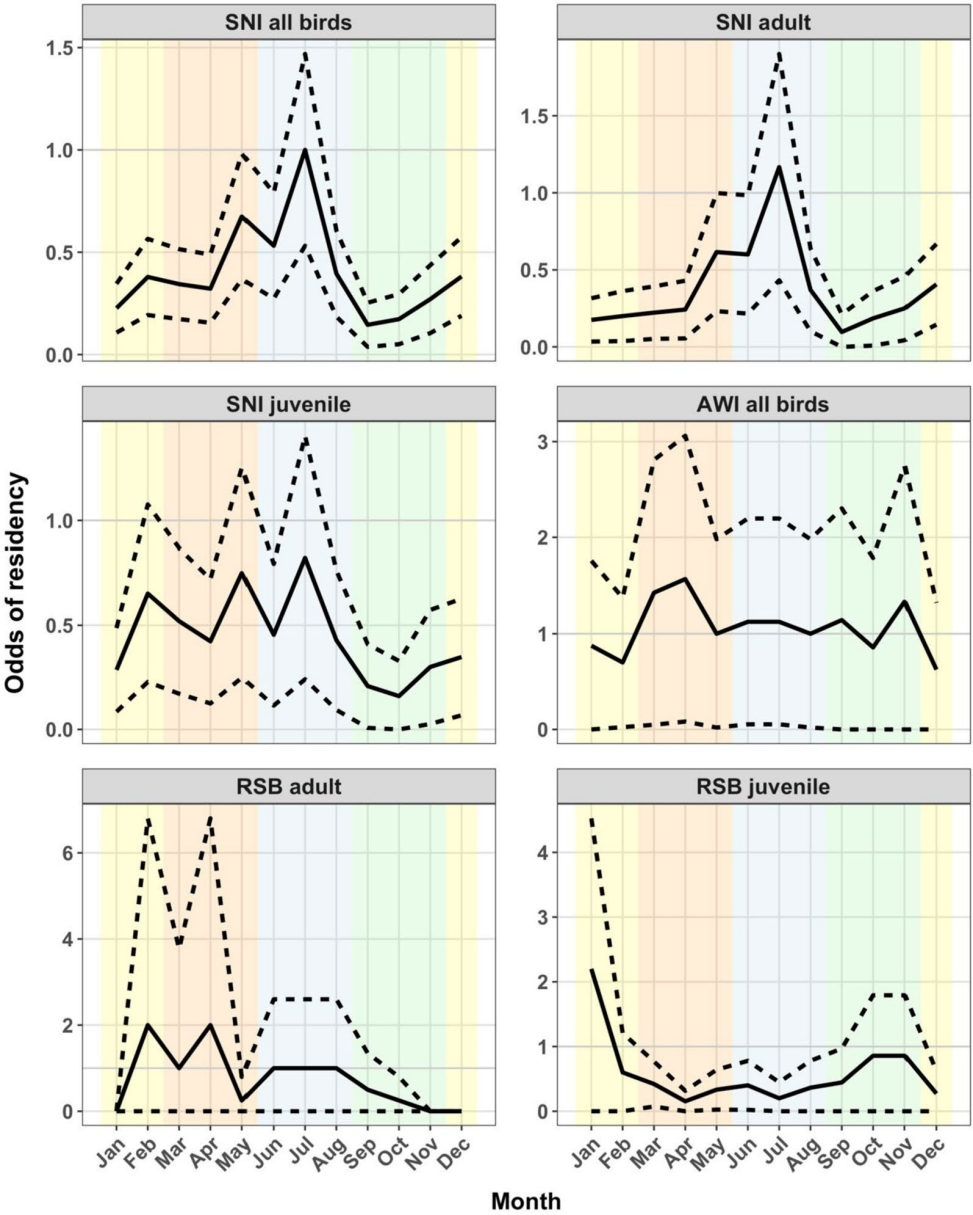
Odds of residency (black line) per calendar month. Dashed lines indicate the upper and lower 95% confidence interval (CI) for the odds. Shaded regions show the season of the year in the southern hemisphere

SNI were most likely to be resident in July, the middle of winter (Fig. 6), with higher proportion of resident months in autumn (0.3) and winter (0.38) with peaks in odds in May and June. The odds of residency were lowest in spring. Two sample permutation tests for differences in proportions of months resident within seasons showed winter proportions were greater than summer and spring (null hypothesis prop.1 = prop.2, prop.winter – prop.summer = 0.38 – 0.24, permutation *P* = 0.001 and prop.winter – prop.spring = 0.38 – 0.16, permutation *P* = 1e^−6^). Autumn also showed evidence of a great proportion than spring (prop.autumn – prop.spring = 0.29 – 0.16, permutation *P* = 7e^−4^). Within age groups, adult SNI showed clearly increased odds of residency during winter (proportion resident months in winter = 0.4, prop.winter – prop.spring = 0.38 – 0.15, permutation *P* = 4e^−5^). Juvenile odds of residency among months were less consistent, with multiple small peaks in late summer, late autumn, and mid-winter and reduced odds in spring. Permutation *P*-values only showed evidence for autumn and winter being more resident than spring (prop.winter – prop.spring = 0.36 – 0.18, permutation *P* = 0.005 and prop.autumn– prop.spring = 0.36 – 0.18, permutation *P* = 0.007) but no evidence for differences between other seasonal proportions for juveniles.

RSB juveniles showed reduced residency in autumn (0.24) and winter (0.31), and increased odds in spring (0.35) and summer (0.39) (Fig. 6). Permutation *P*-values only showed marginal evidence for summer proportions being greater than autumn (prop.summer– prop.autumn = 0.15, permutation *P* = 0.04). Data for adult RSBs were limited but appeared to show no evidence for difference in seasons based on permutation tests for proportions. Juveniles showed similar results to the all-RSB analysis (Fig. 6). AWI odds of residency were relatively consistent through the year, with small peaks in autumn (March–April) and spring (November) but no evidence for differences in proportions of months resident in each of the seasons (Fig. 6).

### Site fidelity

A total of 147 residency utilisation distribution areas (UD areas) were identified, representing areas and periods of time for which individuals roosted and foraged in the same place but were not breeding. Of these, 81 areas were used by multiple individuals (55%). The maximum number of individuals tracked using any one residency area over the whole period of record was 25 (median 2.0; mean 3.9 ± SD. = 4.4), at Barmah-Millewa Forest on the Murray River at the NSW-VIC border. Of the 89 individuals with periods of residency, 33 (37%) had more than one residency area identified. The largest number of residency areas identified for an individual was 7 areas, for an adult SNI. The greatest number of separate residency *periods* identified for an individual was 25, for an adult SNI tracked for > 5 yr. The median time spent visiting any residency area was one day (mean 11.0 ± SD. = 32.0; range < 1—679 days), while the median time between visits was three days (mean 36.0 ± SD. = 126.0; range 1 – 1,263 days). Arrivals and departures from residency areas were most frequent in March (autumn) and least frequent in July (winter; Supplementary Fig. 7). The median number of revisits to a residency area across all birds was six (mean 12.22 ± 18.67; range 1—110). Areas with the highest total revisits across all birds were: Barmah-Millewa Forest; Hird Swamp and Johnson Swamp in the Kerang Lakes area of the Loddon catchment; the adjacent Gunbower–Koondrook–Perricoota Forest area including Kow Swamp; and the Macquarie Marshes (Supplementary Table 4; Supplementary Figs. 8–10).

**Fig. 7:**
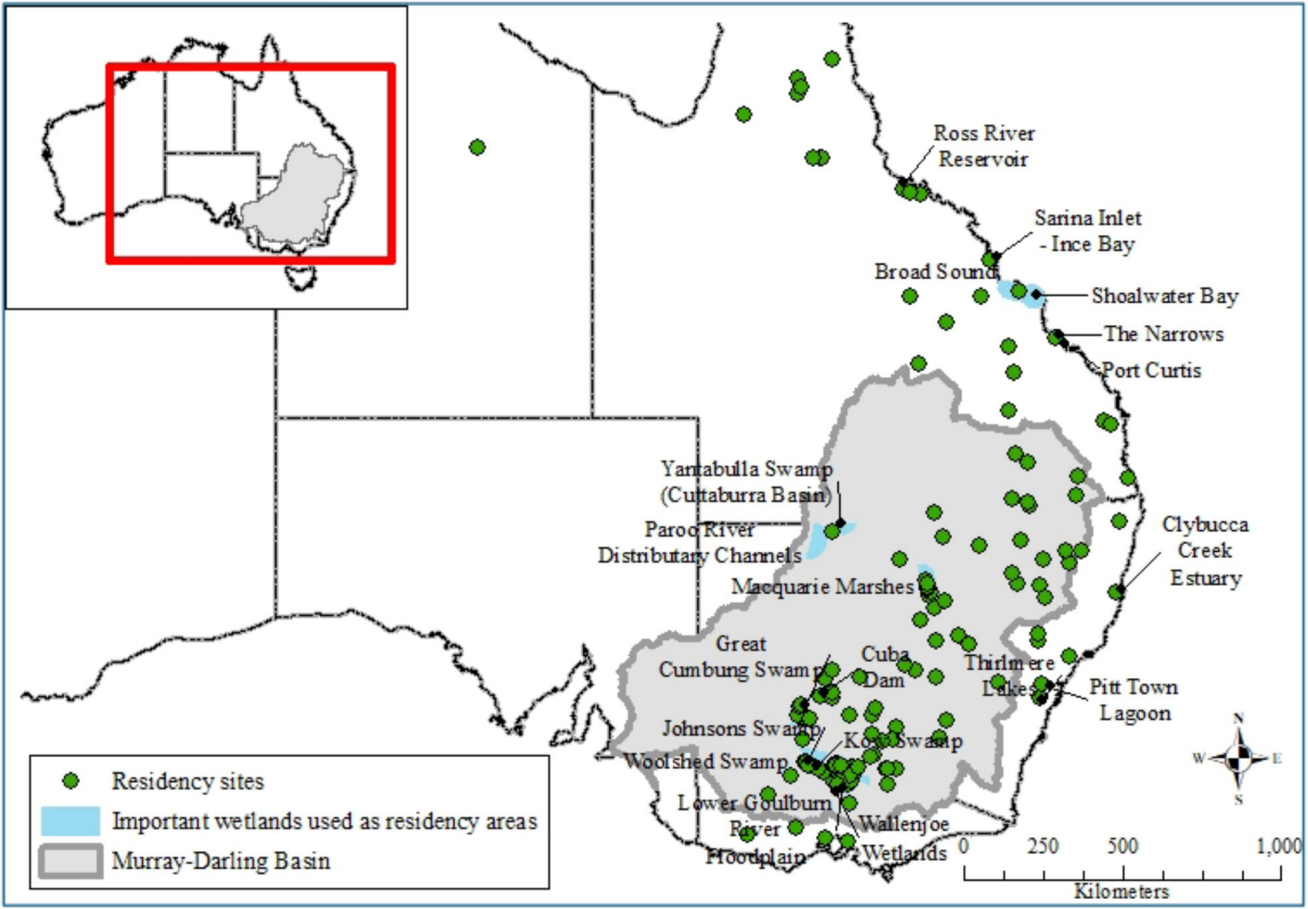
Residency areas for non-nesting ibis and spoonbills, and associated wetlands listed under the Directory of Important Wetlands in Australia, including seven sites listed under Ramsar

Sixty-five percent of the 147 identified residency areas were not associated with wetlands listed nationally or internationally as important. These ‘non-listed’ residency areas were spread across eastern Australia (Fig. 7). There were 27 wetlands listed in the Directory of Important Wetlands in Australia used as residency areas (51 unique UDs; Fig. 7; Supplementary Table 4). Seven of these are listed under the Ramsar convention: Barmah Forest; Millewa Forest and Koondrook–Pericoota Forests (NSW Central Murray State Forests); the Macquarie Marshes; Gunbower Island (Gunbower Forest), Hird Swamp and Johnson Swamp (Kerang Wetlands). These wetlands are also known breeding sites for aggregate-nesting species.

**Fig. 8:**
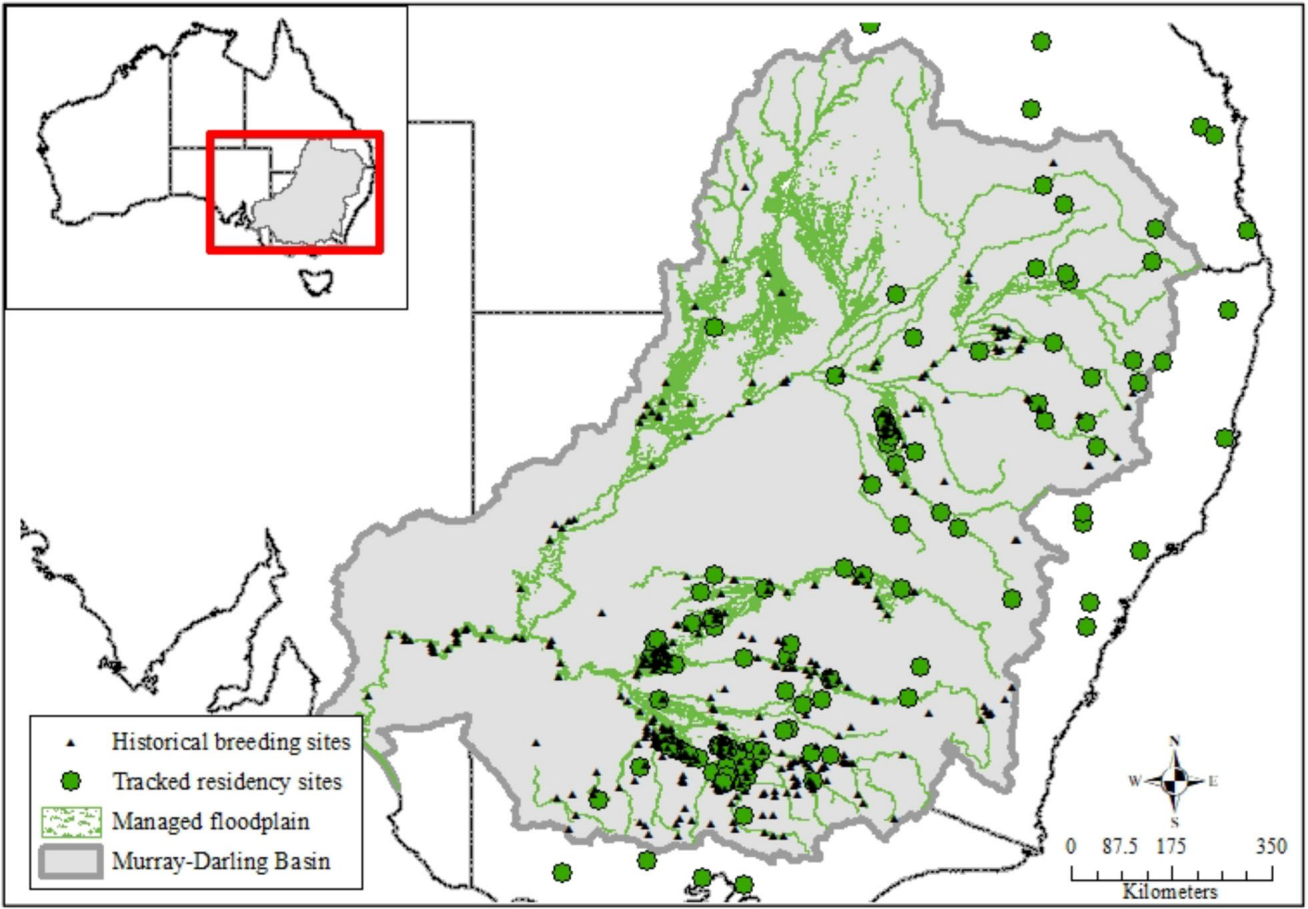
Residency areas for non-nesting ibis and spoonbills mapped with known nesting sites and ‘managed floodplain’ within the Murray-Darling Basin

Seventy-four percent of the residency areas identified were located within the Murray-Darling Basin. Overall, 42 of the 147 residency areas overlapped known breeding wetlands for aggregate-nesting species within the Murray-Darling Basin (29%; Fig. 8; Table 2). Almost half of the 147 residency areas overlapped the ‘managed floodplain’ (72; 49%; Fig. 8), including all the 42 breeding area overlaps.

**Table 2:** Known breeding areas used during residency periods for roosting and foraging within the Murray-Darling Basin

Catchment	Breeding wetland	Number of residency areas (UDs)
Murray	Barmah-Millewa Forest	14
Murrumbidgee	Gayini Floodplains	5
Loddon	Kerang Lakes	4
Macquarie	Macquarie Marshes	4
Goulburn	Goulburn River Floodplains	3
Lachlan	Lower Lachlan Floodplains	3
Broken	Broken Creek Wetlands	2
Murray	Gunbower-Koondrook-Perricoota Forest	2
Lachlan	Cuba Dam	1
Lachlan	Mid Lachlan Floodplains	1
Murrumbidgee	Mid Murrumbidgee River Floodplains	1
Ovens	Ovens River Floodplains	1
Goulburn	Wallenjoe Wetlands	1

## Discussion

Long-term research is important for understanding survival strategies in long-lived species [68, 69]. This study is the first to track multi-year movements of substantial numbers of ibis and spoonbills in Australia using satellite telemetry. From it we have quantified movement distances and timing, residency and non-residency, areas used when resident, and species and individual movement strategies. A range of scales of movement was apparent, from movements of only tens of km over months to movements of hundreds of km through eastern Australia within a few days or weeks.

### Movement strategies within and among species

The tracking results reported here have revealed mixed movement strategies within and among the three species tracked. Each species had individuals displaying plasticity in behaviours across the movement spectrum over time, using different movement strategies at both annual and sub-annual temporal scales [70, 71]. Overall, each species tended toward differences in dominant movement strategy. The dominant strategy for SNI as a species was clearly nomadism, while the dominant strategy for AWI was residency – with the caveat that only seven AWI were tracked. The dominant strategy for RSBs was less clear, but with only 33% of months spent resident by adult spoonbills and relatively low residency odds for juveniles, is also likely to be nomadism.

The odds of residency for juveniles overall were lower than for adults for both SNI and RSB, implying that juveniles are potentially more likely to take advantage of opportunities presented by climate, weather and management actions than adults over monthly to yearly timescales [72] – but also potentially more difficult to support with management in predictable ways at these temporal scales [1, 73]. During periods of residency, the shorter-range movements of juveniles compared to adults in terms of both distances travelled to forage and roost shifts suggest that provision of resources for juveniles should ideally be prioritised at locations close to known roost sites – which is also potentially more difficult for managers, reducing ‘choice’ of watering sites. Despite these potential difficulties, it will remain important to support juveniles wherever possible, in order to support recruitment into the adult breeding population, which for these species can take four years.

**Fig. 9:**
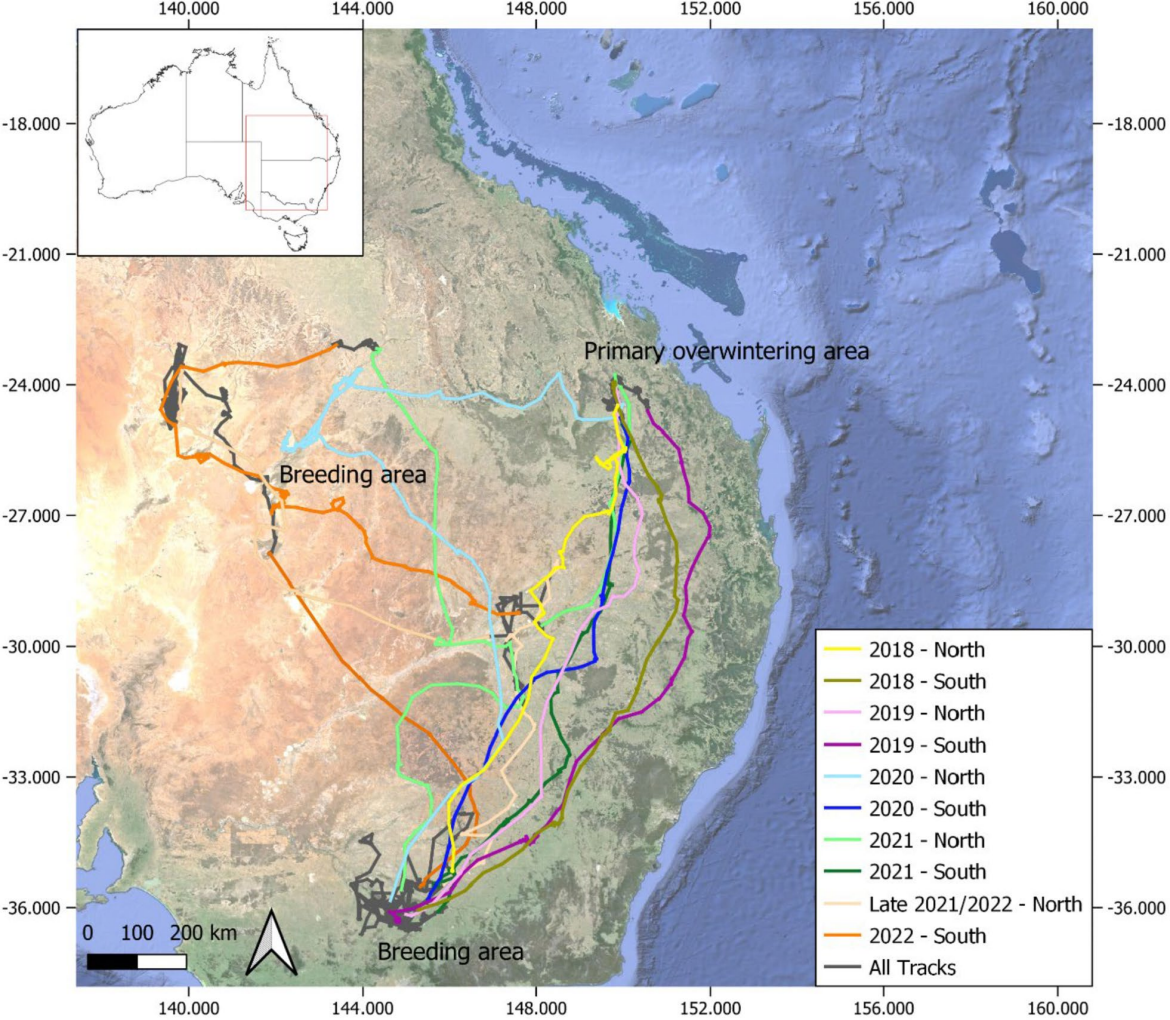
Satellite tracked movements of an adult SNI between December 2017 and October 2022

None of the individuals tracked were classical ‘obligate migrants’ for the entire duration of tracking, but some individuals moved seasonally north–south between the same places in some years and showed some site fidelity. For example, in the first two years of tracking, the movements of one adult SNI suggested that this individual was classically migratory, moving between the same breeding and overwintering sites at about the same time each year (Fig. 9). However, in the third year, this individual diverted from the usual route to the previous overwintering site and instead flew to an area experiencing extensive flooding hundreds of kilometres away. This individual then flew east via a different route to the usual overwintering site, arriving several months later than the previous two years. There was also flexibility in the timing of movements in terms of departures and arrivals, but with this flexibility set within seasonal patterns. For example, individuals that migrated north–south in autumn and spring varied in the precise timing of their movements and were flexible about the routes and time taken to reach their destinations.

### Benefits and vulnerability arising from flexible movement strategies

While the variability in movement strategies documented in this study is known to be common among vertebrates, it remains less well studied than ‘typical’ migratory movements despite indications that such plasticity significantly influences the capacity of species and populations to cope with environmental change [19, 74]. Satellite telemetry studies are increasingly demonstrating that mixed movement strategies are more common in birds than previously thought [18, 33, 75–77]. It has been estimated that > 36% of Australian land bird species may be ‘partially migratory’ [20] and it is likely that this also applies to waterbird species. This is because Australian inland and especially freshwater environments are subject to extreme variability and unpredictability. Plasticity in movement strategies is thought to be a population characteristic that facilitates species persistence in the face of spatial and temporal variation in resources, environmental conditions and their predictability [19, 74, 75, 77, 78]. For such plasticity to persist in a species, there must be benefits to individuals using each strategy when integrated over time [75, 77, 78]. For example, an individual ibis remaining resident near a breeding area through winter may have first choice of prime nesting sites in spring or be able to take advantage of good breeding conditions early. In contrast, an individual moving long distances (either migrating or moving nomadically) may be able to avoid unfavourable weather or resource conditions, particularly in winter, thereby improving body condition and surviving for longer.

A nomadic or strategy-flexible individual may take advantage of relatively unpredictable booms in water and food resources but can move away from unfavourable conditions and choose to return to favoured breeding sites when conditions are suitable. The latter strategy is likely to be the most advantageous when climatic and resource conditions are highly unpredictable and highly variable – as they are in Australia [79–81]. An ability to switch between strategies among years allows the species to deal with climatic and weather variability and associated resource fluctuations [74]; this is particularly the case for SNI due to their broad diet and their ability to successfully forage in terrestrial environments for long periods [40, 43, 82, 83]. This may explain the high proportion of individuals that are classed as ‘non-resident’ but not typically migratory in this study. In the long-term, such a nomadic strategy is likely to allow these species to adapt to climate change better than some other species that are either classically migratory or highly resident and are, therefore, vulnerable to change at particular sites [16, 84]. However, this will differ among species and individual dependence on, or fidelity to, important breeding areas or sites, as suggested for RSBs in New Zealand [85].

While long distance movements and migration can provide benefits, they also have energy costs and can increase the risk of mortality relative to staying resident [86, 87]. This is particularly the case if conditions in ‘resident’ or breeding sites remain good or improve over time, or when conditions elsewhere are becoming more unpredictable or less suitable. In the northern hemisphere, Buchan et al. [75] found evidence in some partially migratory populations of passerine birds, mammals, and reptiles that resident individuals showed greater survival than migratory individuals, counter to author expectations. They and others have suggested that climate change and other anthropogenic changes, such as urbanisation, may be altering conditions to either favour residency or to promote strategic plasticity in birds, depending on the species and situation [18, 88, 89].

This seems likely for AWI, which, even with the low numbers this study tracked, appears to display residency. It has been suggested for this species that the relatively high and reliable resource availability in coastal urban environments, together with a loss of resources inland, may have resulted in a split of some sub-populations from a larger more mobile population [44, 90]. The spread of breeding populations of the AWI into coastal cities, with extensive use of parks and recreation areas, rubbish or landfill sites and areas near airports, has caused conflict with humans [44–46]. Radio-telemetry studies suggest that AWI are largely sedentary in urban environments, with strong site fidelity and most movements occurring within c. 50 km of their breeding or nesting colonies [91]. This suggests reduced habitat and population connectivity between urban populations and inland populations of AWI, similar to that for the American white ibis (*Eudocimus albus*) in Florida USA [92], despite AWI being capable of rapid long-distance movements inland [26, 28]. Some ibis and spoonbill species in the northern hemisphere show evidence of within-species variation in movement strategies geographically, for example the northern bald ibis and Eurasian spoonbill [33, 86, 87], with some suggestion that this is at least partly driven by regional differences in resource availability [93]. Further satellite tracking from a wider range of sites will assist with evaluating such assertions.

In general, the implications of the plasticity of movement strategies documented here for management are that provision of suitable resources for such species must still be undertaken within appropriate seasons according to life cycle stage (e.g. nesting), but the location, timing and duration of such provisions can differ and still have benefits. Effectively, management strategies for these species and their habitats can and should also be ‘plastic’ and adaptable.

### Site fidelity

There is some fidelity to sites with both roosting and foraging habitat, and there are clearly important sites used by multiple individuals and revisited frequently. In addition, while many individual foraging sites used by the three species reported upon here are not recognised by existing conservation or management strategies (such as the Directory of Important Wetlands in Australia or the Ramsar Convention), or are outside of the Murray-Darling Basin and its ‘managed floodplain’, the most heavily used sites generally do fall within these priority management areas. While the true relative importance of non-listed and listed sites for overall population dynamics remains unknown, managers will need to take bird use of non-listed sites into account when evaluating species responses to management actions, such as environmental watering. Some non-listed sites may be of conservation importance for these species, and while environmental watering may be employed in one area, birds may choose to use other sites if conditions are perceived to be better there. A lack of perceived responses to management actions (listing sites, or priority management actions) may arise from such bird responses but not be a true indicator of failure per se.

The availability of food in foraging habitats and their distance from suitable roosting or nesting sites will be critical in determining responses and are potentially manageable. For example, statistics describing distances travelled from roosting sites to foraging sites such as those derived from satellite tracking and described here enable development of threshold distances from roosts within which sites could be prioritised and environmental watering or other management to support foraging areas or food availability could be applied. The degree and timing of residency also allows managers to assess which species or age groups are more likely to be reliant on management of particular sites for long periods or at particular times. Similarly, understanding home-range sizes when resident allows assessment of the area of resources such as environmental watering needed to support species or age groups when resident. It is essential that evaluation of site-based waterbird responses considers these types of contextual information beyond the site, namely, at landscape and whole of basin scales. This is most likely to be the case in relatively wet periods such as La Niña, when natural rainfall and flooding produce vast areas of highly productive foraging and breeding habitats, often long distances from regularly managed wetland sites.

### Implications of large-scale movements

This tracking has revealed high levels of connectivity in eastern Australia for these species, from the south to the north, contrasting with an apparent disconnect between eastern and western populations. The degree and timing of connectivity is likely to be influenced by wind and water conditions as well as by season, as shown for SNI in previous work [94]. This suggests that the lack of species presence records in intervening areas and the lack of recoveries of banded individuals that have moved between eastern and western Australia is most likely because of the extent of arid environments between the latter, rather than because of the scarcity of human observers in these areas, as previously suggested [39, 40, 43]. Within eastern Australia, it is clear that individuals can move long distances quickly and may visit multiple sites within a season over broad areas before selecting a place for temporary residency or nesting. Exploration of potential nesting sites can start in late winter (e.g. August) and can cover hundreds of km for a single individual.

This suggests that managers wishing to provide suitable foraging, refuge or breeding habitat may need to coordinate among sites at these broader spatial scales. For example, at some sites starting watering early, particularly in southern sites where natural flood regimes and cues would have typically started much earlier than in the north, and at other sites starting watering later in the breeding season. These results emphasise the need for multi-scale thinking in planning environmental water allocations for and managing expectations regarding inland waterbird behavioural and population responses. This, together with multiple major breeding sites for these species being in the Murray-Darling Basin of eastern Australia, reinforces the importance of environmental water management for waterbirds at Basin-scale in influencing the maintenance of overall species populations in eastern Australia [52].

## Conclusion

Many waterbird species are highly mobile and move across jurisdictional boundaries and at continental scales, which makes strategic conservation management challenging. Information about individual-, population- and species-level movement strategies, flexibility and plasticity is essential for evidence-based management. For example, prioritisation of sites for environmental watering to support juvenile survival and adult recovery from breeding can be informed by data on site-fidelity, dispersal routes, and foraging sites. Closer examination of ‘non-listed’ sites for which tracked birds have shown site fidelity may reveal areas worthy of additional conservation management. Long-term satellite tracking is a valuable tool in providing this entire lifecycle information and can be used to resolve knowledge gaps, enabling management decisions to be made with improved understanding of waterbird movements and site use.

This is the most detailed study to date of Australian waterbird movements, with 122 individuals tracked between 2016 and 2023. Characterisation of long-term movements provided evidence for variability in movement strategies, including plasticity over an individual’s lifetime. The dominant strategy of our focal species, SNI, was nomadism; however, this varied among individuals, with some evidence for partial migration. We recommend that future research explore potential relationships between bird movements and environmental covariates including weather, water, vegetation, natal site and breeding site. Such relationships that may predict and explain movement strategies as well as finer scale movement responses such as initiation, duration, direction, and cessation of movements in these and related species. Such information would help to explain the cues that birds are responding to at a range of scales and to predict likely responses to management actions. Overall, increased knowledge of the interaction of waterbirds with their environment across their entire life cycles will be increasingly important for informing policy and management decisions and predictions aimed at increasing waterbird abundance and maintaining waterbird diversity.

## Supplementary Information

Additional file 1.

## Data Availability

The data supporting the conclusions of this article are available on reasonable request to the corresponding author. Data and code will be published via the CSIRO Data Access Portal: https://data.csiro.au/
