# Supplementary material for: Satellite telemetry reveals complex mixed movement strategies in ibis and spoonbills of Australia: implications for water and wetland management
Source: Mov Ecol. 2024 Nov 26;12:74. doi: 10.1186/s40462-024-00515-4 (PMC11590462; doi:10.1186/s40462-024-00515-4)
Supplement: Supplementary file 1 — Additional file 1. [file 40462_2024_515_MOESM1_ESM.docx]

# Supplementary information

Supplementary Table 1 Summary statistics for number of days tracked per bird. AWI = Australian white ibis; RSB = royal spoonbill; SNI = straw-necked ibis.

| **Species** | **Age class** | **Minimum** | **Quartile 1** | **Median** | **Mean** | **Standard deviation** | **Quartile 3** | **Maximum** | **n**  **(birds)** |
| --- | --- | --- | --- | --- | --- | --- | --- | --- | --- |
| **AWI** | **Adult** | 156 | 499.5 | 843 | 687 | 472 | 952 | 1062 | 3 |
| **AWI** | **Juvenile** | 301 | 522 | 920 | 1093 | 855 | 1492 | 2233 | 4 |
| **RSB** | **Adult** | 98 | 256 | 424 | 401 | 229 | 552 | 675 | 5 |
| **RSB** | **Juvenile** | 32 | 55 | 111 | 196 | 189 | 332 | 709 | 37 |
| **SNI** | **Adult** | 32 | 109 | 235 | 421 | 464 | 519 | 1844 | 45 |
| **SNI** | **Juvenile** | 41 | 120 | 297 | 460 | 528 | 523 | 2233 | 28 |

Supplementary Table 2 Summary statistics for maximum distance travelled (km) from roost per day. AWI = Australian white ibis; RSB = royal spoonbill; SNI = straw-necked ibis.

| **Species** | **Age class** | **Minimum** | **Quartile 1** | **Median** | **Mean** | **Standard deviation** | **Quartile 3** | **Maximum** | **n**  **(birds)** |
| --- | --- | --- | --- | --- | --- | --- | --- | --- | --- |
| **AWI** | **Adult** | 0 | 0 | 1 | 4 | 16 | 4 | 267 | 3 |
| **AWI** | **Juvenile** | 0 | 1 | 2 | 4 | 8 | 4 | 171 | 4 |
| **RSB** | **Adult** | 0 | 1 | 2 | 9 | 28 | 6 | 415 | 5 |
| **RSB** | **Juvenile** | 0 | 1 | 2 | 8 | 28 | 6 | 546 | 37 |
| **SNI** | **Adult** | 0 | 1 | 2 | 13 | 40 | 6 | 580 | 45 |
| **SNI** | **Juvenile** | 0 | 1 | 2 | 8 | 27 | 4 | 484 | 28 |

Supplementary Table 3 Residency area (km^2^) statistics by species and age class.

| **Species** | **Age class** | **Min** | **2.5 percentile** | **Mean** | **Median** | **SD.** | **97.5 percentile** | **Max** | **N Blocks** | **N** |
| --- | --- | --- | --- | --- | --- | --- | --- | --- | --- | --- |
| **AWI** | **Adult** | 0.01 | 0.1 | 9.3 | 10 | 8.8 | 23.5 | 25.1 | 7 | 3 |
| **AWI** | **Juvenile** | 2.1 | 3.0 | 76.9 | 34.4 | 113 | 320 | 347 | 11 | 4 |
| **RSB** | **Adult** | 0.01 | 0.8 | 83.3 | 55.5 | 91 | 245 | 257 | 8 | 4 |
| **RSB** | **Juvenile** | 0.01 | 0.0 | 43.2 | 11.4 | 55.8 | 169 | 198 | 42 | 31 |
| **SNI** | **Adult** | 1.0 | 1.9 | 84.4 | 49.7 | 120 | 415 | 608 | 53 | 26 |
| **SNI** | **Juvenile** | 0.01 | 0.9 | 46.2 | 15.5 | 68.6 | 265 | 302 | 40 | 24 |


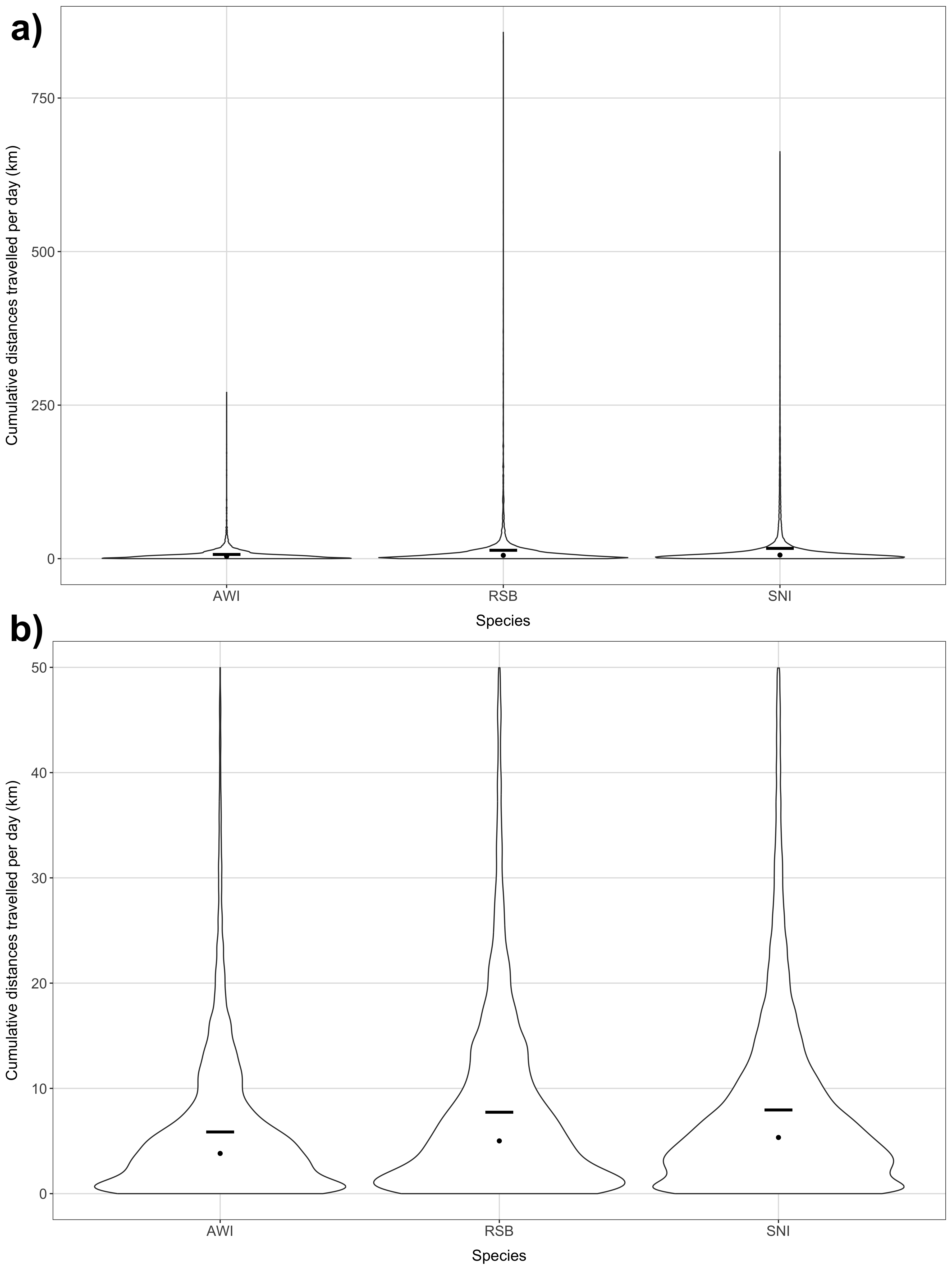


Supplementary Figure 1 Violin plot of the of cumulative distance travelled per day by species. Panel a) shows the total distribution and b) a truncated depiction focussing on the distributions for values less than 50. The cross bar in each of the distributions for each species shows the mean and the point the median.


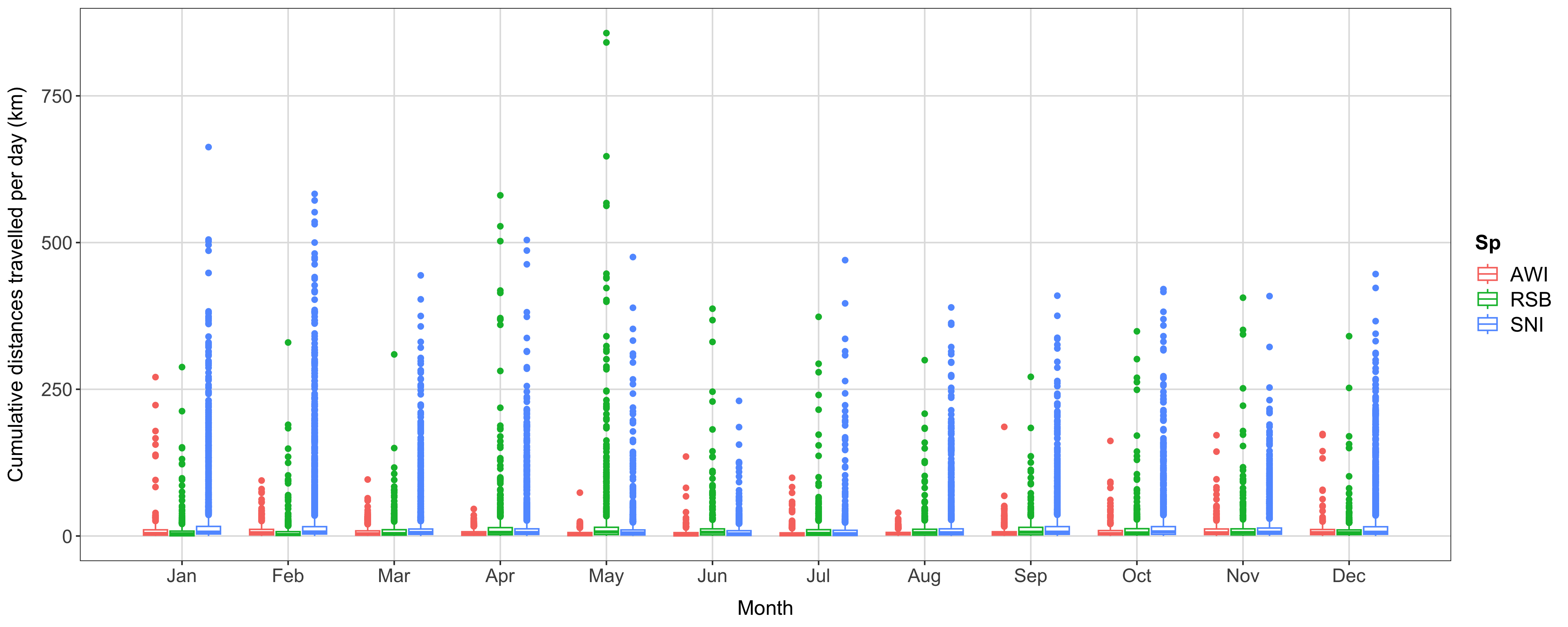


Supplementary Figure 2 Boxplot summaries of the of cumulative distance travelled per day by species within month. Plots shows the right-skewed nature of the cumulative daily distances.


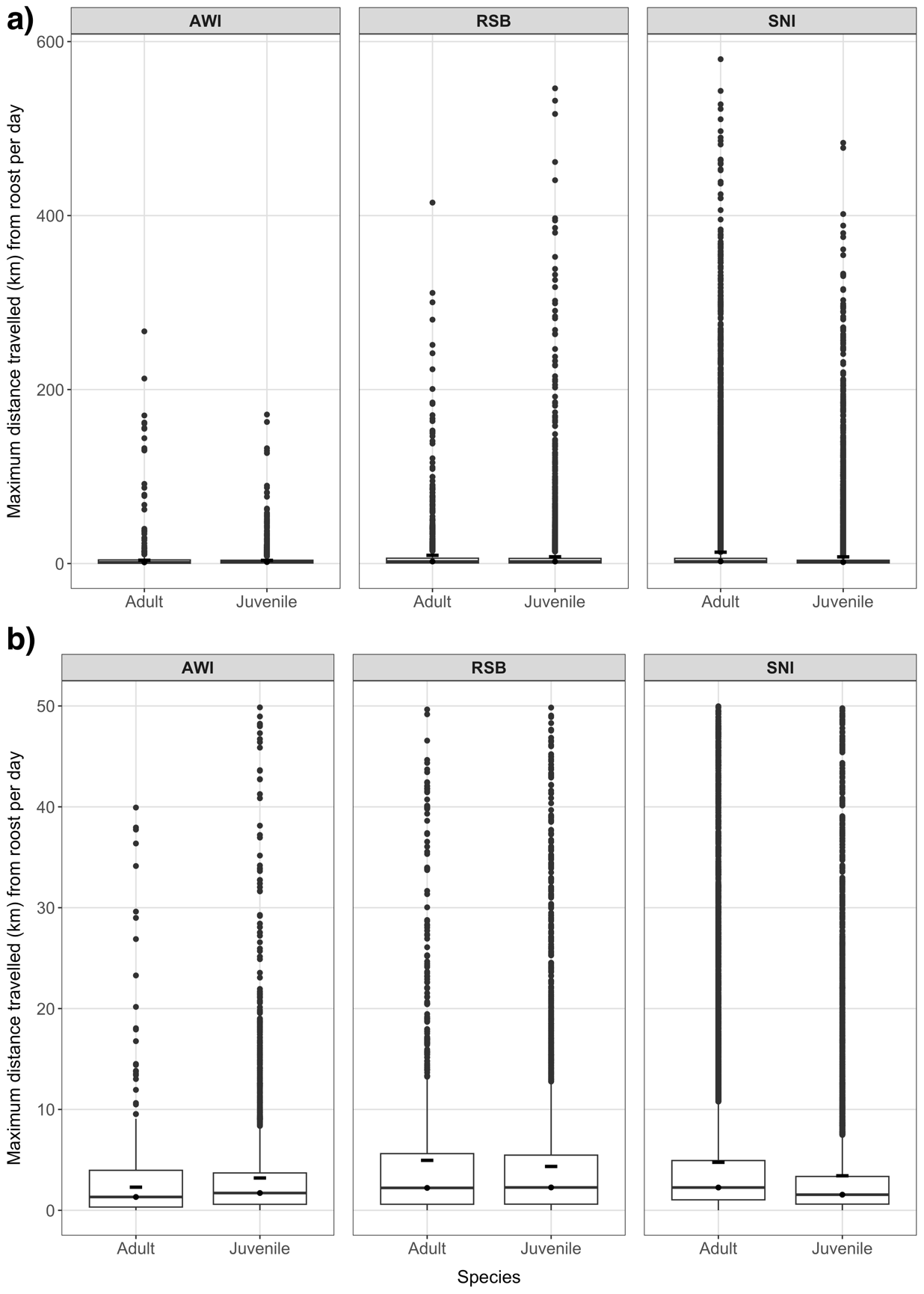


Supplementary Figure 3 Boxplot plot of the of maximum distance travelled from roost per day by species. Panel a) shows the total distribution and b) a truncated depiction focussing on the distributions for values less than 50 km. The cross bar in each of the distributions for each species shows the mean and the point the median.


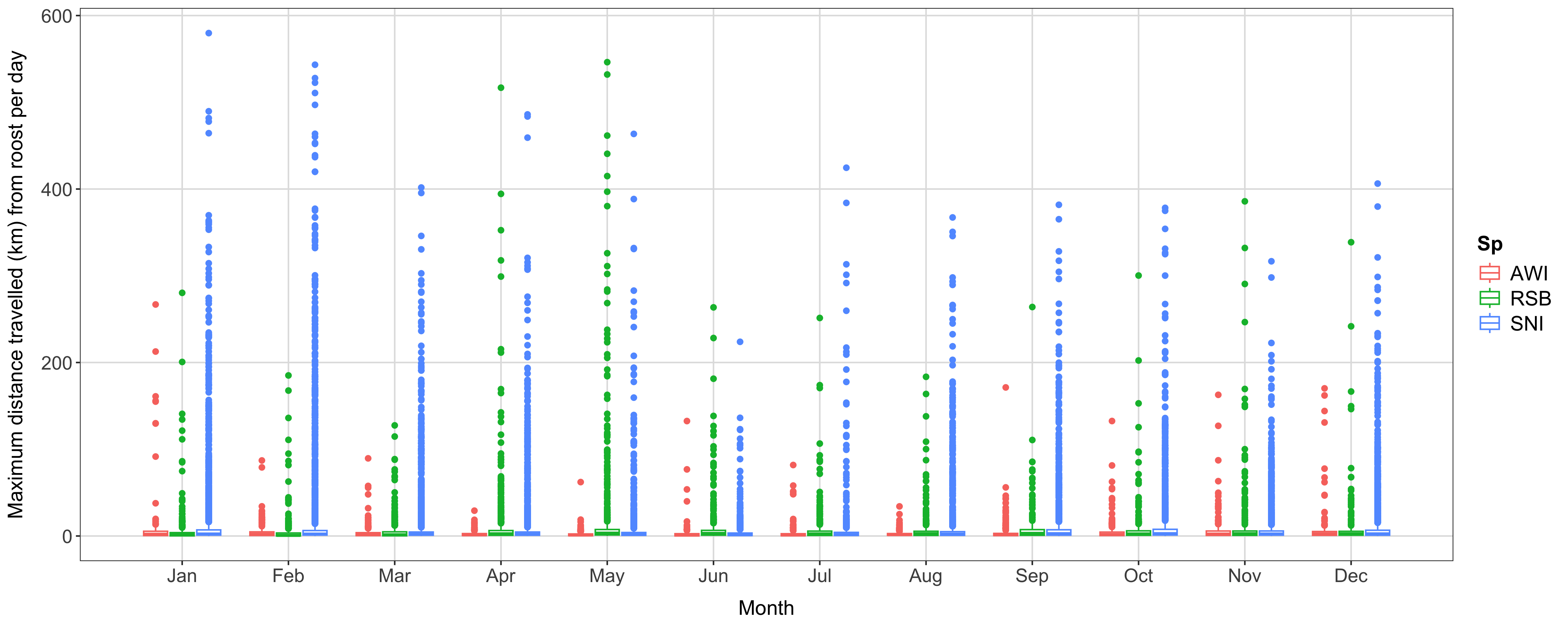


Supplementary Figure 4 Boxplot summaries of the of maximum distance travelled from roost per day by species within month. Plots shows the right-skewed nature of the cumulative daily distances.


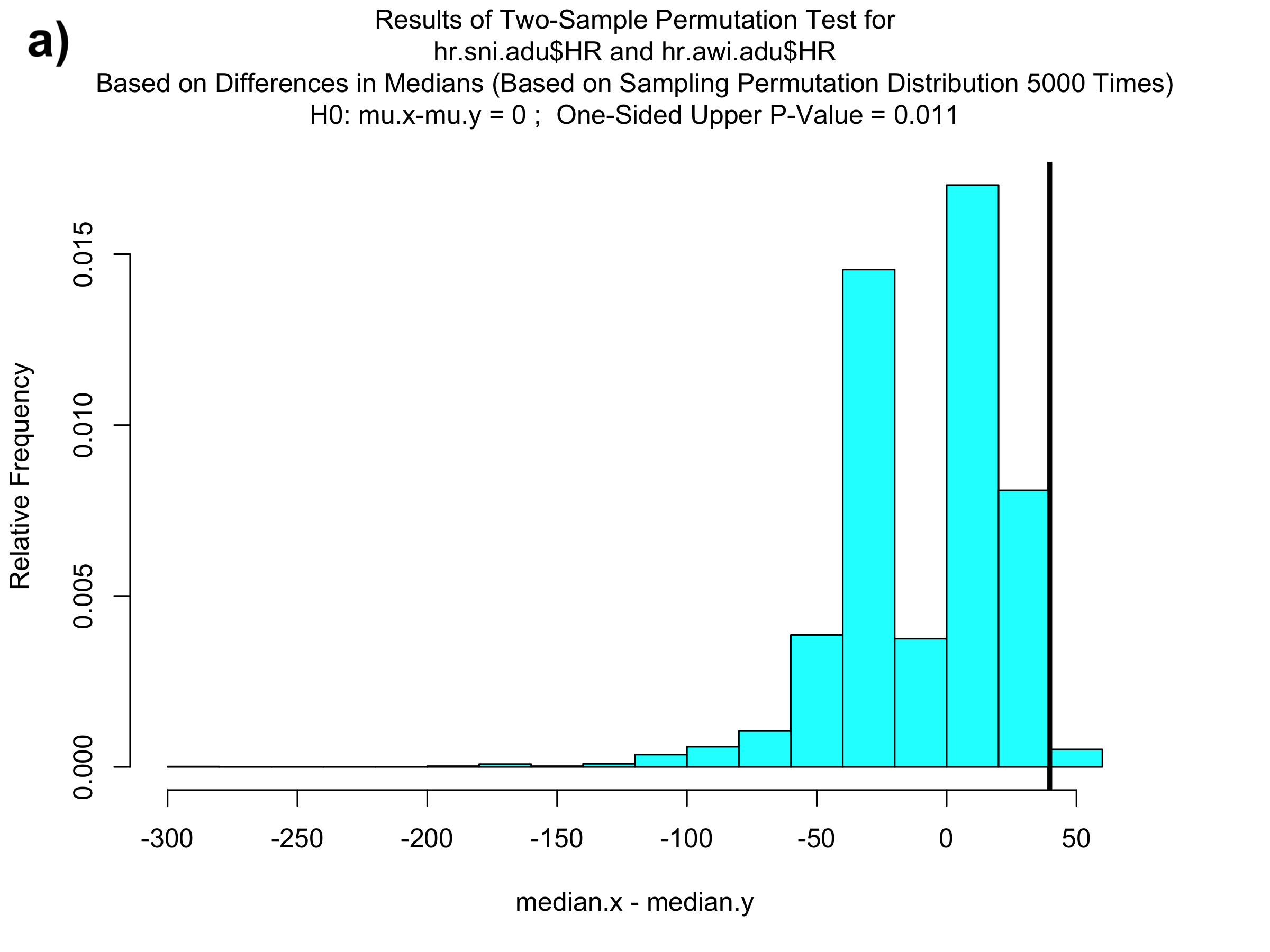


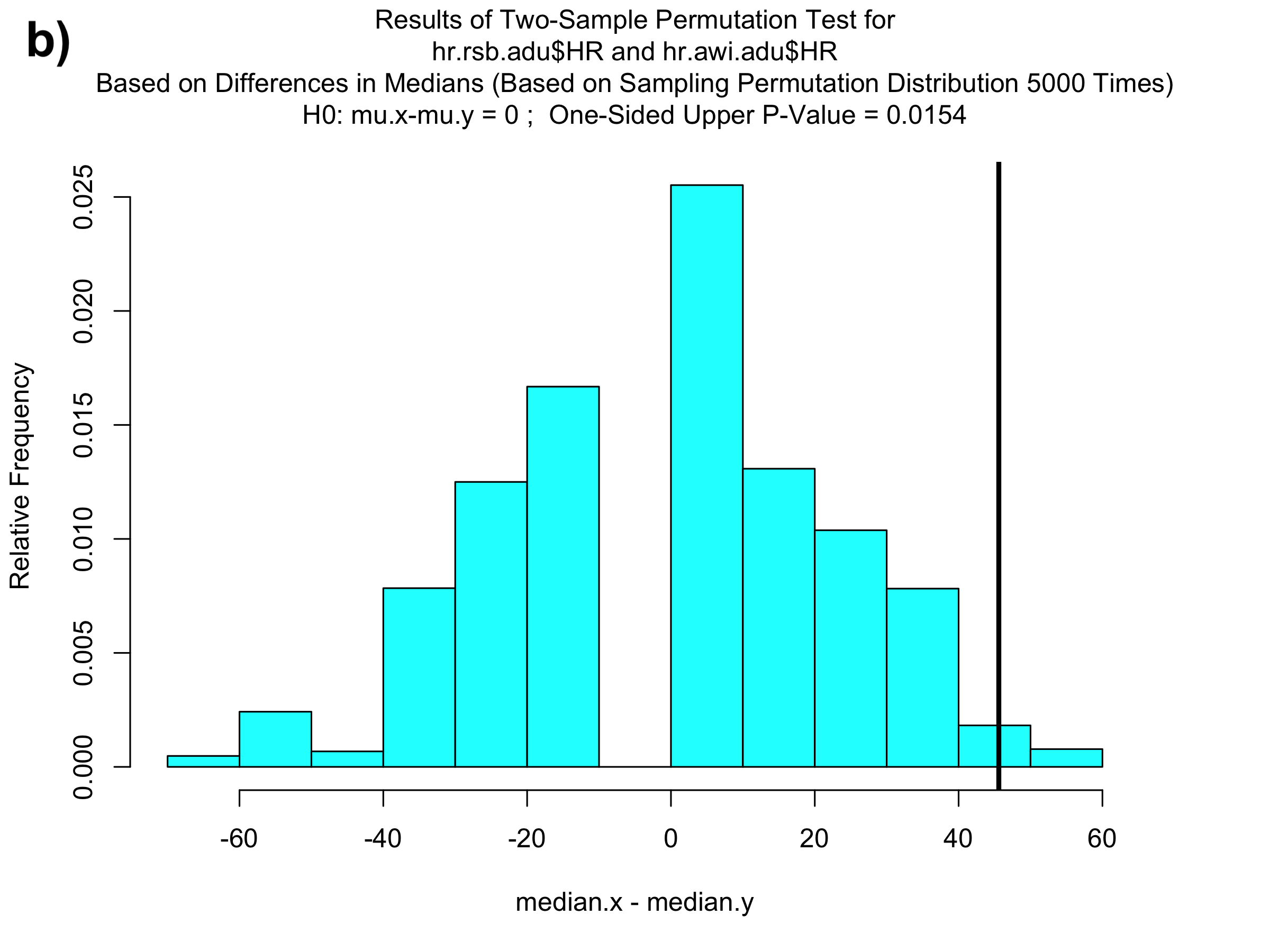


Supplementary Figure 5 Permutation test results from differences of median 95% residency areas between a) adult Australian white ibis (AWI) and straw-necked ibis (SNI,) and b) Australian white ibis (AWI) and royal spoonbills (RSB,).


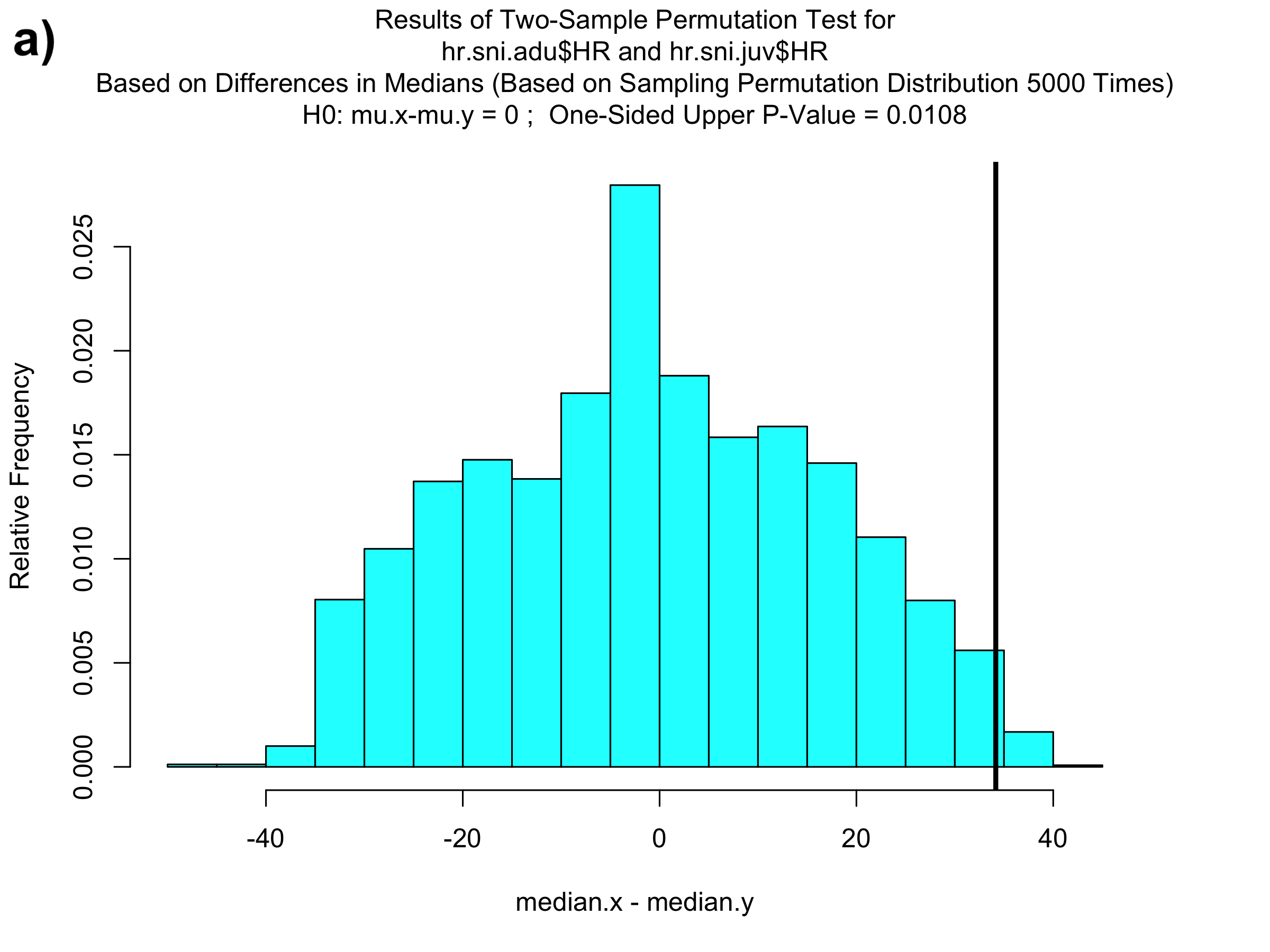

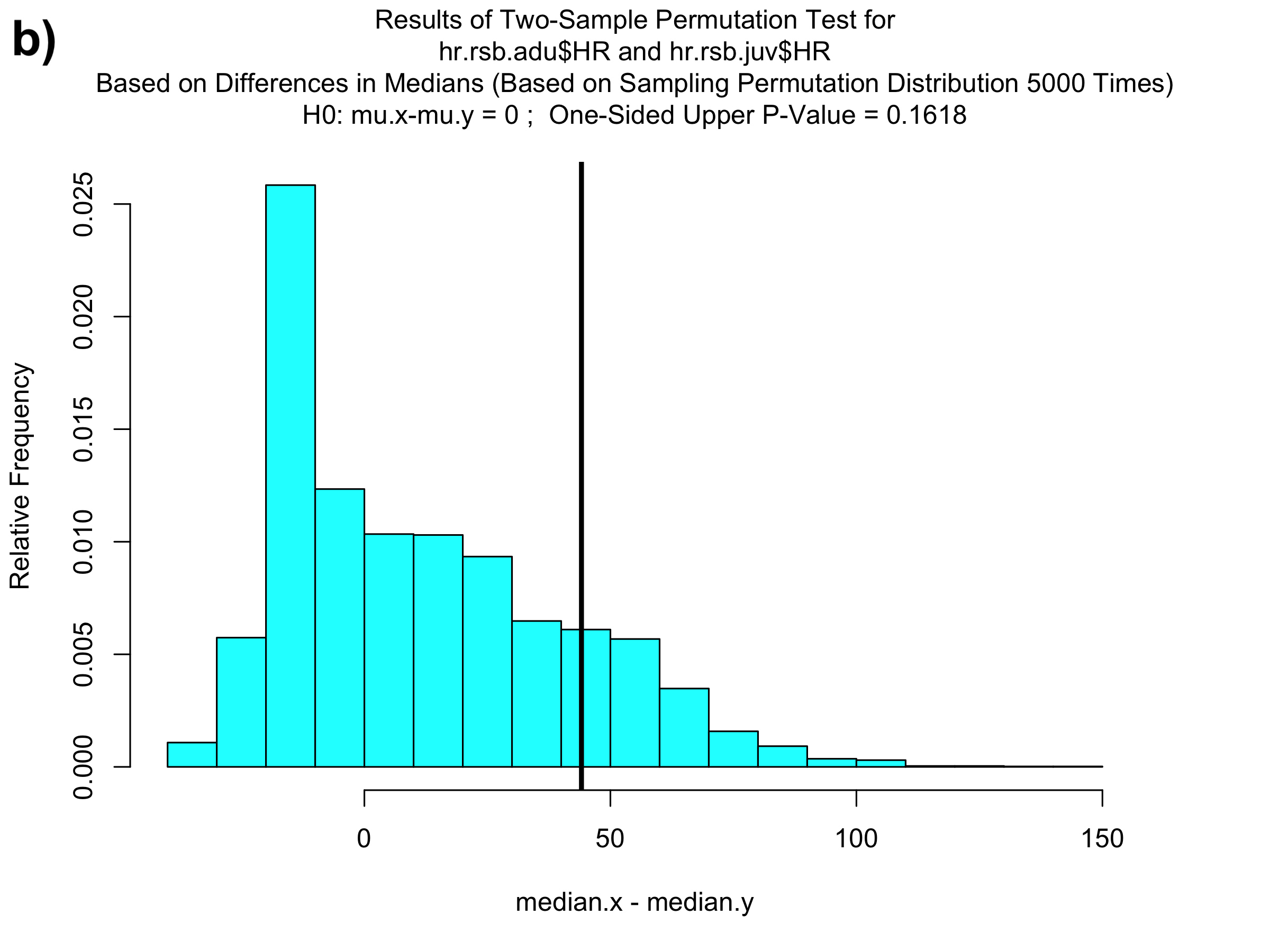


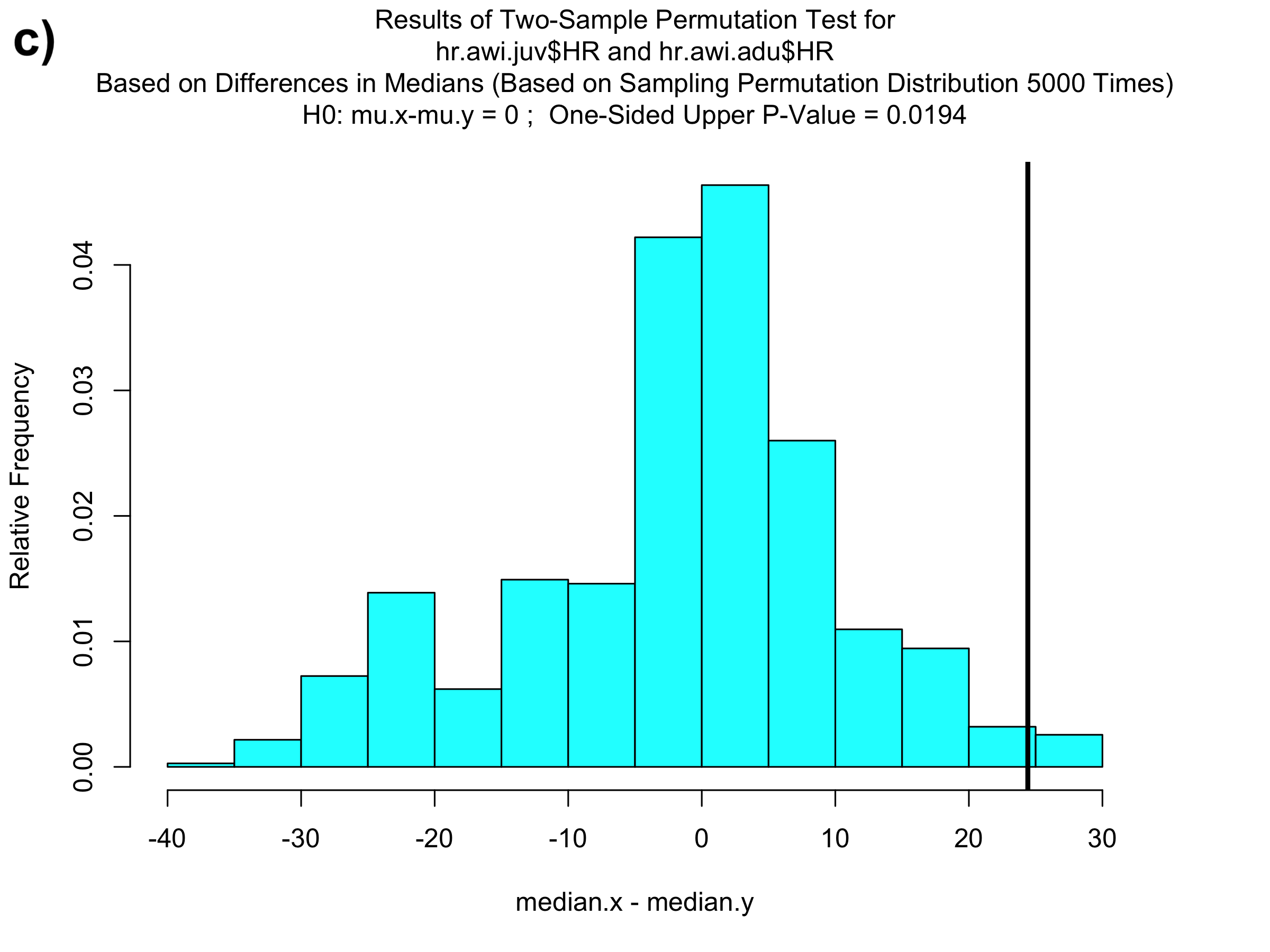


Supplementary Figure 6 Permutation test results from differences of median 95% residency areas between adult and juvenile straw-necked ibis (a), royal spoonbill (b), and Australian white ibis (c). Blue histogram shows the permuation distribution and the vertical bar the observed differences in medians.

Supplementary Figure 7 Total number of arrivals and departures from residency areas by month over all birds.


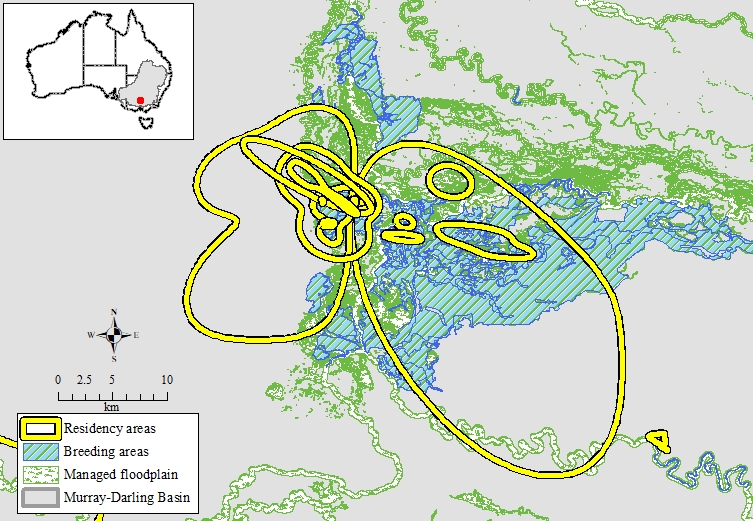


Supplementary Figure 8 Residency areas from many birds intersecting the managed floodplain and breeding areas in and around Barmah-Millewa Forest. The yellow polygons represent the maximum likelihood estimate of the 95% AKDE contours for the area of each bird’s residency block in that location.


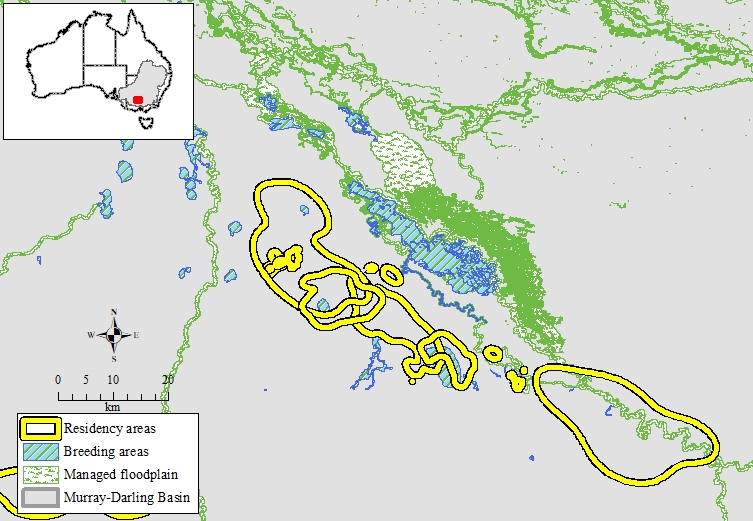


Supplementary Figure 9 Residency areas intersecting the managed floodplain and breeding areas in and around Johnson Swamp, Hird Swamp, Kow Swamp, and the Gunbower-Koondrook-Perricoota Forests. The yellow polygons represent the maximum likelihood estimate of the 95% AKDE contours for the area of each bird’s residency block in that location.


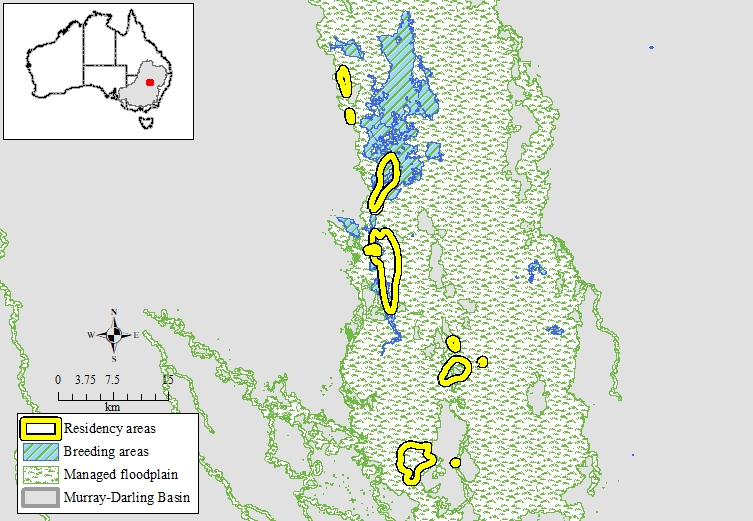


Supplementary Figure 10 Residency areas intersecting the managed floodplain and breeding areas in and around the Macquarie Marshes. The yellow polygons represent the maximum likelihood estimate of the 95% AKDE contours for the area of each bird’s residency block in that location.

Supplementary Table 4 Utilisation distribution areas and bird visits to Ramsar wetlands and sites listed in the Directory of Important Wetlands in Australia.

| **Sites** **listed in the Directory of Important Wetlands in Australia** | **Number of UDs** | **% UDs** | **Number of visits (all birds)** | **Ramsar name (wetland name)** |
| --- | --- | --- | --- | --- |
| Millewa Forest | 13 | 48 | 347 | NSW Central Murray State Forests (Millewa State Forest Group) |
| Macquarie Marshes | 8 | 30 | 139 | The Macquarie Marshes (Macquarie Marshes Nature Reserve) |
| Lower Goulburn River Floodplain | 4 | 15 | 96 |  |
| Barmah-Millewa Forest | 3 | 11 | 103 | Barmah Forest |
| Broken Creek | 3 | 11 | 92 |  |
| Gunbower Island | 3 | 11 | 105 | Gunbower Forest |
| Hird's Swamp | 2 | 7 | 217 | Kerang Wetlands (Hird Swamp) |
| Johnson's Swamp | 2 | 7 | 217 | Kerang Wetlands (Johnson Swamp) |
| Kow Swamp | 2 | 7 | 124 |  |
| Broad Sound | 1 | 4 | 6 |  |
| Clybucca Creek Estuary | 1 | 4 | 1 |  |
| Cuba Dam | 1 | 4 | 16 |  |
| Great Cumbungi Swamp | 1 | 4 | 7 |  |
| Koondrook and Perricoota Forests | 1 | 4 | 31 | NSW Central Murray State Forests (Koondrook Forest Group) |
| Lowbidgee Floodplain | 1 | 4 | 61 |  |
| Merrowie Creek (Cuba Dam to Chillichil Swamp) | 1 | 4 | 16 |  |
| Paroo River Distributary Channels | 1 | 4 | 9 |  |
| Pitt Town Lagoon | 1 | 4 | 2 |  |
| Port Curtis | 1 | 4 | 3 |  |
| Ross River Reservoir | 1 | 4 | 2 |  |
| Sarina Inlet - Ince Bay Aggregation | 1 | 4 | 3 |  |
| Shoalwater Bay Training Area Overview C | 1 | 4 | 6 |  |
| The Narrows | 1 | 4 | 10 |  |
| Thirlmere Lakes | 1 | 4 | 2 |  |
| Wallenjoe Wetlands | 1 | 4 | 12 |  |
| Woolshed Swamp | 1 | 4 | 3 |  |
| Yantabulla Swamp (Cuttaburra Basin) | 1 | 4 | 9 |  |
